# Supplementary material for: Using Patient-Reported Outcome Measures (PROMs) to promote quality of care and safety in the management of patients with Advanced Chronic Kidney disease (PRO-trACK project): a mixed-methods project protocol
Source: BMJ Open. 2017 Jun 30;7(6):e016687. doi: 10.1136/bmjopen-2017-016687 (PMC5726096; doi:10.1136/bmjopen-2017-016687)
Supplement: Author's manuscript [file bmjopen-2017-016687.draft_revisions.pdf]

BMJ Open

Using Patient-Reported Outcome measures (PROMs) to promote quality of care and safety in the management of patients with Advanced Chronic Kidney Disease (PRO-trACK Project) – A mixed-methods project protocol

Journal:	*BMJ Open*
Manuscript ID	bmjopen-2017-016687
Article Type:	Protocol
Date Submitted by the Author:	02-Mar-2017
Complete List of Authors:	Aiyegbusi, Olalekan; University of Birmingham, Institute of Applied Health Research; University of Birmingham, Centre for Patient-Reported Outcomes Research (CPROR)<br>Kyte, Derek; University of Birmingham, Institute of Applied Health Research; University of Birmingham, Centre for Patient-Reported Outcomes Research (CPROR)<br>Cockwell, Paul; Queen Elizabeth Hospital Birmingham, Department of Renal Medicine; University of Birmingham, Centre for Patient-Reported Outcomes Research (CPROR)<br>Marshall, Tom; University of Birmingham, Institute of Applied Health Research; University of Birmingham, Centre for Patient-Reported Outcomes Research (CPROR)<br>Dutton, Mary; Queen Elizabeth Hospital Birmingham, Department of Renal Medicine<br>Slade, Anita; University of Birmingham, Institute of Applied Health Research; University of Birmingham, Centre for Patient-Reported Outcomes Research (CPROR)<br>Marklew, Neil; University of Birmingham, Patient Advisory Group Member, Centre for Patient-Reported Outcomes Research (CPROR)<br>Price, Gary; University of Birmingham, Patient Advisory Group Member, Centre for Patient-Reported Outcomes Research (CPROR)<br>Verdi, Rav; University of Birmingham, Patient Advisory Group Member, Centre for Patient-Reported Outcomes Research (CPROR)<br>Waters, Judi; University of Birmingham, Patient Advisory Group Member, Centre for Patient-Reported Outcomes Research (CPROR)<br>Sharpe, Keeley; University of Birmingham, Patient Advisory Group Member, Centre for Patient-Reported Outcomes Research (CPROR)<br>Calvert, Melanie; University of Birmingham, Institute of Applied Health Research; University of Birmingham, Centre for Patient-Reported Outcomes Research (CPROR)
**Primary Subject Heading**:	Patient-centred medicine
Secondary Subject Heading:	Health services research, Qualitative research, Renal medicine, Research methods
Keywords:	NEPHROLOGY, Chronic renal failure < NEPHROLOGY, QUALITATIVE RESEARCH, STATISTICS & RESEARCH METHODS, USABILITY TESTING,

	VALIDATION STUDY

SCHOLARONE™ Manuscripts

For peer review only

Using Patient-Reported Outcome measures (PROMs) to promote quality of care and safety in the management of patients with Advanced Chronic Kidney Disease (PRO-trACK Project) – A mixed-methods project protocol

Olalekan Lee Aiyegbusi[1, 2], Derek Kyte[1, 2*], Paul Cockwell[1, 3], Tom Marshall[1, 2], Mary Dutton[3], Anita Slade[1, 2], Neil Marklew[4], Gary Price[4], Rav Verdi[4], Judi Waters[4], Keeley Sharpe[4], Melanie Calvert[1, 2]

1. Centre for Patient-Reported Outcomes Research, University of Birmingham, Edgbaston, Birmingham, UK

2. Institute of Applied Health Research, University of Birmingham, Edgbaston, Birmingham, UK

3. Department of Renal Medicine, University Hospitals Birmingham NHS Foundation Trust, Queen Elizabeth Hospital Birmingham, Mindelsohn Way, Edgbaston, Birmingham, UK

4. Patient Advisory Group Member, Centre for Patient-Reported Outcomes Research, University of Birmingham, UK

Email: Dr O.L. Aiyegbusi, oxa238@bham.ac.uk - Dr D. Kyte, d.g.kyte@bham.ac.uk - Prof P. Cockwell, paul.cockwell@uhb.nhs.uk - Prof T. Marshall, t.p.marshall@bham.ac.uk - M. Dutton, Mary.Dutton@uhb.nhs.uk - Dr A. Slade, A.L.Slade@bham.ac.uk - Prof M. Calvert, m.calvert@bham.ac.uk

*** Correspondence**: Derek Kyte, d.g.kyte@bham.ac.uk

Word count: 4,052

For peer review only

ABSTRACT

Introduction: Advanced chronic kidney disease (CKD) has a major effect on the quality of life and health status of patients and requires accurate and responsive management. The use of electronic patient reported outcome measures (ePROMs) could assist patients with advanced pre-dialysis CKD, and the clinicians responsible for their care, by identifying important changes in symptom burden in real time. We report the protocol for Pro-trACK, which will explore the feasibility and validity of an ePROM system for use in patients with advanced CKD.

Methods and analysis: The project will utilise a mixed-methods approach in three studies. These will comprise: (i) patient usability testing of the ePROM system focusing on acceptability and technical performance/stability; (ii) ascertaining the views of patient and clinician stakeholders on the optimal use and administration of the CKD ePROM system - this will involve qualitative face-to-face/telephone interviewing and/or focus groups with: patients, clinical staff, management and IT team members; (iii) psychometric assessment of the system, within a cohort of patients with advanced CKD, to establish the measurement properties of the ePROM.

Ethics and dissemination: This project was approved by the West Midlands Edgbaston Research Ethics Committee (Reference 17/WM/0010).

The findings from this project will be provided to clinicians at the Department of Renal Medicine, Queen Elizabeth Hospitals, Birmingham (QEHB), NHS England, presented at conferences and to the Kidney Patients' Association, British Kidney Patient Association and the British Renal Society. Articles based on the findings will be written and submitted for publication in peer-reviewed journals.

For peer review only

Strengths and limitations of this project

- Whilst there is evidence to support the use of ePROMs in the management of other conditions, notably cancer, the evidence for the use of ePROMs in the management of patients with CKD is currently limited. The PRO-trACK project will help fill this evidence gap.
- By using a mixed methods approach, the project will provide a rigorous exploration of the acceptability, validity and feasibility of the ePROM system for the management of patients with CKD.
- This project will only involve patients with CKD stages 4 and 5 and patients on dialysis for $\leq$ 6 months as the ePROM intervention is presently intended for patients with advanced CKD stages 4 and 5.

For peer review only

INTRODUCTION

Chronic kidney disease (CKD) is a general term that refers to a number of disorders that affect the structure and function of kidneys.[1] The definition of CKD is based on sustained reduction in renal function (i.e. "estimated glomerular filtration rate (eGFR) <60 mL/min per 1·73 m² for 3 months or more") and/or evidence of structural or functional abnormalities of the kidneys regardless of clinical diagnosis.[1 2] CKD is associated with other long-term conditions such as hypertension, cardiovascular diseases and diabetes will increase the risk of ill health both when present singly and when associated with other long-term conditions.

CKD causes clinical signs and symptoms, particularly when the disease is advanced.[3] The most commonly experienced are fatigue, drowsiness, pain, pruritus and dry skin.[4] These symptoms often occur concurrently and may negatively affect patients' daily activities and their physical, emotional and psychological well-being.[5] Therefore impacting on the quality of life of (QOL) of those affected, particularly as the disease progresses towards end-stage renal disease.[6 7]

The symptoms of CKD progression can be monitored using self-completed questionnaires known as patient-reported outcome measures (PROMs) which capture information about health status from patients' own point of view.[8] Although commonly administered in paper format, PROMs can be completed as electronic patient-reported outcome measures (ePROMs) using multiple digital platforms. This makes it possible to remotely monitor patients and generate 'real time' data about patient symptoms and QOL. As patients with advanced CKD are at risk of deteriorating rapidly and developing cardiovascular complications,[9] the use of an ePROM system may help clinicians detect deterioration of symptoms and assist with

For peer review only

the tailoring of treatment to the needs of each patient.[10-12] Health-related issues that matter to patients may also be identified using ePROM data and this could potentially facilitate communication and shared-decision making between patients and their clinicians.[13-15] In stable patients, the use of ePROMs may reduce the occurrence of unnecessary clinical appointments.[12]

In Denmark the WestChronic ePROM System has been successfully implemented for tailoring the care of various patient groups[12], while in the UK, patients with cancer have been successfully monitored for the side effects of chemotherapy using the ePROM Advanced Symptom Management System (ASyMS).[16] However, there is limited information on the use of ePROMs in the management of adult patients with CKD in a routine clinical setting. Therefore, the aim of the project is to explore the feasibility and validity of an electronic patient-reported outcome measure (ePROM) system for monitoring and assisting with the individual management of patients with advanced CKD.

Description of the ePROM system

The ePROM system will be designed as an electronic method of allowing patients with CKD to remotely self-report their symptoms and quality of life using a digital platform that is convenient to them (PC, tablet, smartphone, telephone voice recognition or scanned paper copy), providing important patient-centred data to the patients' clinical team. A patient advisory group (PAG) met prior to commencing the project and considered the acceptability, burdensomeness and relevance of four questionnaires for the target CKD group. Three questionnaires were selected based on this consultation and these will be electronically adapted and tested in the ePROM system.

For peer review only - http://bmjopen.bmj.com/site/about/guidelines.xhtml

The ePROM system will be accessed via the secure electronic patient portal developed by the University Hospitals Birmingham NHS Foundation Trust, known as 'myhealth@QEHB' (See Figure 1).[17] myhealth@QEHB currently has 14,000 patient users and was awarded the prestigious E- Health Insider award in 2014.[18]

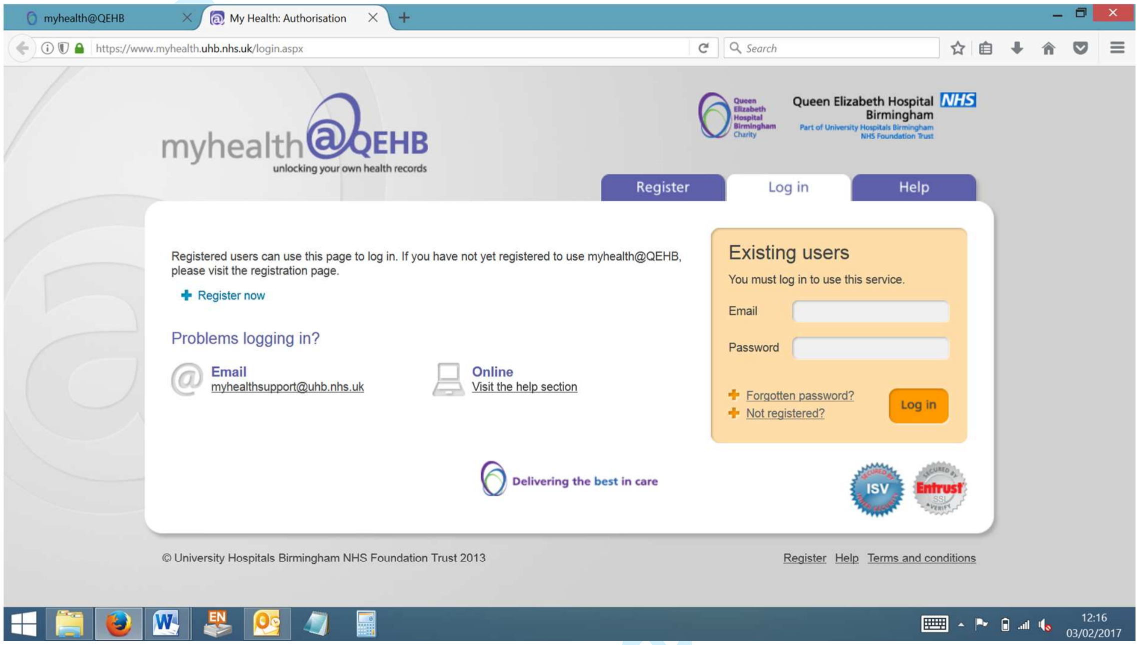

Fig. 1. Screenshot of the myhealth@QEHB login page.

For peer review only - http://bmjopen.bmj.com/site/about/guidelines.xhtml

Overview of the PRO-trACK Project

The PRO-trACK project will consist of 3 studies namely:

a) Usability testing: Usability testing refers to the appraisal of a product or service by potential service users and involves the observation of such users completing a task within a predetermined scenario.[19 20] The ePROM system will be tested by patients with CKD stages 4 and 5.

b) Qualitative study: This will explore the views of stakeholders on the optimal use and administration of the CKD ePROM system. This will involve (1) qualitative face-to-face/telephone interviews with patients with CKD and (2) focus groups/interviews with clinical staff from the QEHB renal service and members of the myHealth team and hospital management staff, as required.

c) The validation of the ePROM system: The purpose of this study is to evaluate the measurement properties of the electronic versions of the selected PROMs hosted on the 'myhealth@QEHB' server. At the end of this study, the most suitable questionnaire (s) would be taken forward for formal feasibility testing.

Research objectives

Study 1 – Usability testing

i. To determine whether patients can easily navigate the ePROM system.

ii. To determine if patients are able to complete the 3 questionnaires successfully on their own, and if not, how much assistance they require.

iii. To determine the average length of time required to complete an ePROM report.

For peer review only

iv. To determine the level of satisfaction with the ePROM interface.

v. To identify changes that might be required to improve user performance and satisfaction.

Study 2 – Qualitative Study

i. To determine which symptoms patients with CKD find most bothersome.

ii. To explore how acceptable ePROMs are.

iii. To determine how often patients will be willing to complete the ePROM.

iv. To determine the preferred method of completing the ePROM i.e., PC, smartphone, tablet, telephone voice recognition or paper completion.

v. To explore the likely factors that may improve or discourage the completion of ePROMs.

vi. To explore how they would like to receive feedback from the clinical team regarding the ePROMs they provide.

vii. To evaluate and rate the relevance of the items of the 3 ePROM questionnaires with clinical staff (content validation).

viii. To determine those factors that may improve or discourage the use of ePROM data by clinicians.

ix. To determine clinicians preferred method of displaying ePROM data.

Study 3 – ePROM Validation

i. To determine the reliability and validity of the 3 ePROM questionnaires.

ii. To determine the ability of the ePROM questionnaires to detect change in a patient's health over a period of time.

For peer review only

iii. To determine which of the 3 questionnaires is most suitable to take forward for formal feasibility testing.

For peer review only

METHODS AND ANALYSIS

Project Design

In this section, the project setting and eligibility criteria for participants will be described first as this will be the same for the three studies. Aspects of research methods specific to each study will be subsequently discussed separately.

Project Setting

Queen Elizabeth Hospitals, Birmingham (QEHB) will be the host site for this project. Clinical staff at the Renal Unit, QEHB and academic researchers at the Centre for Patient Reported Outcomes Research, University of Birmingham will be responsible for the conduct and management of the project. The nephrology service comprises 21 consultants, 30 junior doctors, and 20 nurses and allied health professionals in the CKD team.

Project Participants

Patient participants

For the entire project, we will recruit adult patients with advanced CKD stages 4 and 5 under the care of the renal services at QEHB, with eGFR $\leq$ 20ml/min/1.73m^2 and a projected risk of progression to ESRD of >20% within 2-years using the renal risk calculator.[21] They must have been counselled about treatment modalities for end-stage renal disease (ESRD). ESRD is the term used when the eGFR falls below 10 mL/min per 1·73 m².

For peer review only

We have selected this group of patients as our main target for this project because, even though they have not yet reached ESRD, they have the highest symptom burden and a high risk of rapid clinical deterioration to ESRD. We hypothesise that this group of patients are therefore likely to derive the most benefit from an ePROM system.

Patients who have commenced dialysis within 6 months will also be eligible to participate as we hypothesise that they will be able to recall their symptoms and medical needs pre-dialysis. Participants will be required to converse in everyday English and provide informed consent.

Patients who have a recent history of acute kidney injury within the last 3 months, a co-morbidity with a high level of symptoms or terminal illness likely to lead to the death within 6 months of participation will be excluded from the study.

Clinicians and other professional staff

Clinicians who manage patients with CKD at the Renal Unit, QEHB and members of the myHealth team and hospital management staff who provide consent will be recruited for this project.

Recruitment methods

A member of the renal research team at QEHB will screen patients for eligibility using the electronic screening tools that are utilised for clinical purposes. This will identify patients that meet the eligibility criteria and the clinics they attend. Eligibility will be confirmed by direct review of the clinical records by the research nurse and a clinician who are members of the renal care team, on the delegated duty log.

For peer review only

Patients will then be approached at clinic by a member of the renal care team and given patient information sheet to read. Further information about the study will be given and their immediate queries will be addressed. They will be contacted no earlier than 48 hours after the clinic visit to ascertain if they wish to participate in the study.

Clinicians and other professional staff will be recruited by the members of the research team and sent information sheets via email.

For peer review only

Study 1 – Usability testing

Data collection

The scenario for this study will be the self-report of a patient's health status between clinic appointments using an electronic device such as a smartphone, tablet or PC/laptop. Each patient will undergo a single one-to-one session with (OLA) the project's Chief Investigator (CI), and attempt to complete the 3 electronic questionnaires with as little assistance as possible.

The Concurrent Think Aloud (CTA) and Retrospective Probing (RP) moderating techniques will be used for this study.[22] CTA involves the thinking aloud and vocalisation of participants' thoughts during the session while RP refers to interviewing the participant following the completion of the session.[22] The advantage of combining both techniques is that real time feedback of the test session could be obtained which the CI could explore afterwards.[19]

The CI will take detailed notes of the participants' comments, actions, non-verbal cues and errors and pass minimal comments to encourage them to think aloud during the sessions.

Qualitative data will be collected in form of a brief audio-recorded interview at the end of each test. The patients will be questioned based on the notes taken by the CI during the session. They will be encouraged to provide any recommendation to improve user experience.

For peer review only

Data analysis

Thematic analysis will be conducted on the qualitative data (See the analysis of patient interviews for more details), which will include the notes taken during the sessions and the transcripts of the post-test interview.

Quantitative data will be summarised using descriptive statistics such as proportions, averages, percentages and rates.[19] Quantitative data will include successful completion rates, error-free rates and average time required for completion.

Sample size

We will recruit up to 30 patients from QEHB for this study based on the recommendations found in literature.[23] The process of improving the usability of any system is an iterative one;[19] therefore patients will be randomly enrolled into groups of 3 - 5 patients per group. The usability testing with each group will correspond to a test cycle. The findings from each test cycle will guide the process of improving the ePROM system before re-testing in the subsequent cycle. A minimum of 2 test cycles will be conducted for this phase of the study.

For peer review only

Study 2 – Qualitative Study

Study 2a - Patient Interviews

Data collection

A qualitative research method utilising semi-structured interviews will be employed in order to obtain the views and opinions of patients on the use of the ePROM system as part of their care. Face-to-face interviews will be arranged to either coincide with patients' scheduled clinic visit or held on a separate day if preferred. The option of a telephone interview will also be given.

A topic guide will be used to provide a general direction for each interview and ensure that important issues are covered whilst allowing enough flexibility to capture other relevant themes that may be arise during any session.

Interviews will be recorded using an encrypted digital audio recorder and transcribed by a professional transcription company.

Data analysis

The transcripts will be analysed by the CI using the Nvivo 10 software package by QSR International. Thematic analysis of the data will be conducted following the six steps described by Braun and Clarke.[24] The process of analysis will begin with the CI 'actively' reading and engaging with the data set (i.e. searching for patterns and meanings). The next phase will be the initial coding of the raw transcript data using the QSR software. Extracts will be coded inclusively (i.e. a little surrounding data will be kept to ensure that contextual meaning is not lost).[25]

For peer review only

Phase 3 will involve the analysis and organisation of codes into potential themes. These initial themes will be revised and refined in the 4th phase on two levels. The first is at the level of coded data extracts to ensure that they are coherent for each theme. The second level of analysis is to ensure that the themes reflect the data set. During this phase codes and themes that are redundant may be removed, revised or merged as required. Phase 5 will involve the definition of what each theme is and what it is not; and its importance in relation to the entire data and the research questions. The themes will be considered individually as well as in relation to other themes to ensure that overlaps are kept to a minimum. The final phase will be the production of the study report.

The project team (Independent of the CI) will randomly review a sample of transcripts for verification purposes.

Data analysis will be carried out simultaneously with data collection and both will continue until no new themes emerge from the further analysis i.e. data saturation has been reached.[26]

Respondent validation will be undertaken, whereby a summary of the main points arising from the interview will be sent to each participant for comments.

For peer review only

Sample size

A significant proportion of patients with CKD managed by the Renal Unit are of ethnic minority backgrounds. Therefore, patients will be purposively sampled and efforts will be made to recruit eligible minority ethnic patients in order to ensure that the study reflects the diversity that exists within the patient population. Based on experience from previous similar qualitative studies conducted by the research team,

recruitment will continue until a target sample size of between 15 and 30 patient participants is attained or until saturation is achieved.

For peer review only

Study 2b - Focus groups with clinicians and other professional staff

Data collection

Focus group discussions will be held with clinicians who manage patients with CKD at the Renal Unit, QEHB, members of the myHealth team and hospital management staff as required. The purpose of these focus groups will be to evaluate the content validity of the selected questionnaires and discuss the various issues that might encourage or discourage the use of ePROMs.

An independent member of staff will serve as chief moderator and direct the discussions while the CI will act as assistant moderator, making notes and observing the interactions within the groups. The discussions will be allowed to develop with minimum interference following a topic guide to ensure that all the main points are covered. Focus group sessions will be recorded using an encrypted digital audio recorder and transcribed by a professional transcription company.

Face-to-face or telephone interviews with clinicians and other professional staff may be conducted to explore their views on the use of PROMs. These interviews will require a maximum of 1 hour.

Sample size

We will aim to include up to 15 participants in up to three focus groups (5 participants in each group) and if necessary interview the same number of participants.

Data analysis

Thematic analysis will be conducted (See analysis of patient interviews for details).

For peer review only

Study 3 – ePROM Validation

Data collection

Patients will be registered on 'myhealth@QEHB' in order to access the ePROM system. There will be the option of completing paper versions if preferred. Time for questionnaire completion may be influenced by patients' symptoms but should require no more than 1 hour.

All participants will be asked to complete the 3 questionnaires 3 times: at study entry, at 2 weeks after initial completion and at 6 months after initial completion. Completing the questionnaires at these time points will facilitate the comprehensive assessment of psychometric properties.[8]

Data analysis

Quantitative data will be analysed using statistical software such as STATA. Where appropriate, analysis will be conducted separately for patients with CKD stages 4 and 5 and patients on dialysis.

A disclaimer statement will be included in the patient information sheets (PIS) for the validation study informing the patients that the questionnaires will not be assessed until the end of the study; therefore, patients should inform their clinician (e.g. general practice or renal services) of any health care needs for management.

Specific analyses (for definition of measurement properties see Table 1)

Internal consistency reliability: Using baseline scores, Cronbach's alpha will be calculated for the total scales and subscales of the 3 questionnaires. An 'if item

For peer review only

deleted' analysis will be conducted to identify whether any items should be dropped from the scale.

Test–retest reliability: The completion of the ePROMs, 2 weeks after the initial completion will allow the assessment of the stability of the questionnaires. Intra-class correlation coefficients (ICC) will be calculated on subscale and total scores.

Measurement error: Measurement error will be calculated using the standard error of the measurement (SEM). Also the minimally important clinical change (MIC) and the smallest detectable change (SDC) will be calculated. The measurement error and the MIC will help determine which questionnaire can be used to monitor changes in individual patients in routine clinical practice.

Convergent validity: Pearson correlation coefficients will be used to assess the correlations of the scores obtained from the administration of the ePROMs. Correlations between subscale scores will be explored where appropriate.

Structural validity: Confirmatory factor analysis of baseline scores will be used to confirm the item clusters and support the evidence obtained for internal consistency of the ePROMs.

Responsiveness: The ePROMs will be administered 6 months after the initial completion in order to assess the ability of the questionnaires in detecting change in patients' condition. Comparisons will also be made between the scores obtained at these points and routinely collected clinical and laboratory data.

Rasch Analysis: This analysis will be performed to statistically assess the suitability of the 3 questionnaires for the patients and help identify redundant items that could be subsequently removed. It might also provide a more precise estimate of measurement error.

For peer review only

Sample size

We aim to recruit at least 180 patients based on the recommendations found in contemporary literature outlining psychometric best-practice.[27]

For peer review only

Reimbursement and Withdrawal

Reimbursement: All patients will be reimbursed for their time with a £20 gift card.[28 29] Light refreshments will be provided for focus group participants.

Withdrawal: Patient participants will be informed that they have the right to freely withdraw from the study, for any reason, at any time prior to their data being integrated into the data set. They will not be required to supply a reason for their withdrawal and the decision will have no effect on their future medical care.

As focus group participants (clinicians and other professional staff) will be audio recorded as a group, they will be informed that it will be impossible to withdraw their data during or after a focus group discussion. They will not be required to supply a reason for their withdrawal before a focus group discussion and the decision will have no effect on their employment.

For peer review only

DISCUSSION

PROMs can be completed electronically making it possible to remotely monitor symptoms in patients with CKD and generate ‘real time’ data which may assist clinicians with the tailoring of treatment to the needs of each patient.[12] The use of ePROMs could potentially foster patient-clinician communication and further support shared-decision between patients with CKD and their clinicians.[13 14] The regular completion of ePROMs may decrease the need for stable patients with acceptable ePROM data to attend clinical appointments thus sparing them the financial burden and physical stress of travelling. This might free up appointment times for patients that actually need to be seen in clinic.[12] In this manner, the use of PROMs may significantly improve the quality of life of patients with CKD.

At each stage of the project, when necessary, PAG meetings will be convened for their input and drafts of publication manuscripts reviewed by members of the group before submission to journals.

By employing a mixed methods approach, the PRO-trACK project will provide evidence of the feasibility and validity of the ePROM system in patients with advanced CKD.

For peer review only

ETHICS AND DISSEMINATION

Ethics and data management

This project was approved by the West Midlands Edgbaston Research Ethics Committee (Reference 17/WM/0010).

Participant data (whether in electronic or paper format) will be acquired, anonymised, transferred and stored according to the Data Protection Act 1998[30]; the Confidentiality - NHS Code of Practice[31]; the Caldicott principles[32]; the University of Birmingham Code of practice for research[33] and the University of Birmingham Guidance on Out of Hours Activities and Lone Working.[34]

Only members of the research team will have access to the project data. The exception will be permissions given to authorised regulatory personnel in order to conduct audits and inspections on behalf of the ethics committee.

Dissemination

The findings of the project will be provided to the Informatics Team and the Nephrology Unit at the QEHB as required. Participants will be given a summary of the findings, with reference to the full reports if desired.

Research article(s) based on the findings of the studies will be written and submitted for publication to peer-reviewed journals and all contributors and their contributions to the study will be acknowledged.

For peer review only

Acknowledgement

The authors thank all the members of the Patient Advisory Group (PAG), Centre for Patient-Reported Outcomes Research (CPROR), University of Birmingham, for their comments and suggestions during the development of this project.

Author Contributions

MC is the guarantor for this project. The project was conceived by MC, DK, PC and TM and designed by MC, DK, PC, TM, OLA, MD and AS. OLA drafted the protocol manuscript. The manuscript was reviewed by MC, DK, PC, TM, OLA, MD, AS, NM, GP, RV, JW and KS. The final draft approved by all authors.

Funding

This project is funded as part of the Health Foundation's Improvement Science Programme. The Health Foundation is an independent charity working to improve the quality of healthcare in the UK. The Health Foundation was not involved in any other aspect of the project.

Tom Marshall is partly funded by the National Institute for Health Research (NIHR) through the Collaborations for Leadership in Applied Health Research and Care for West Midlands (CLAHRC-WM). This paper presents independent research and the views expressed in this publication are not necessarily those of the NIHR, the Department of Health, NHS Partner Trusts, University of Birmingham or the CLAHRC WM Management Group.

Competing interests: None declared.

For peer review only

Ethics approval: This project was approved by the West Midlands Edgbaston Research Ethics Committee (Reference 17/WM/0010).

Table 1. Definitions of domains, measurement properties, and aspects of measurement properties			
**Domain**	**Measurement property**	**Aspect of measurement property**	**Definition**
**Reliability**			The degree to which the measurement is free from measurement error
**Reliability (extended definition)**			The extent to which scores for patients who have not changed are the same for repeated measurement under several conditions: for example, using different sets of items from the same HR-PROs (internal consistency), over time (test-retest) by different persons on the same occasion (inter-rater) or by the same persons (i.e., raters or responders) on different occasions (intra-rater)
	Internal consistency		The degree of the interrelatedness among the items
	Reliability		The proportion of the total variance in the measurements which is because of "true"[a] differences among patients
	Measurement error		The systematic and random error of a patient's score that is not attributed to true changes in the construct to be measured
**Validity**			The degree to which an HR-PRO instrument measures the construct(s) it purports to measure
	Content validity		The degree to which the content of an HR-PRO instrument is an adequate reflection of the construct to be measured
		Face validity	The degree to which (the items of) an HR-PRO instrument indeed looks as though they are an adequate reflection of the construct to be measured
	Construct validity		The degree to which the scores of an HR-PRO instrument are consistent with hypotheses (for instance with regard to internal relationships, relationships to scores of other instruments, or differences between relevant groups) based on the assumption that the HR-PRO instrument validly measures the construct to be measured
		Structural validity	The degree to which the scores of an HR-PRO instrument are an adequate reflection of the dimensionality of the construct to be measured
		Hypotheses testing	Idem construct validity
		Cross-cultural validity	The degree to which the performance of the items on a translated or culturally adapted HR-

For peer review only

Table 1. Definitions of domains, measurement properties, and aspects of measurement properties			
**Domain**	**Measurement property**	**Aspect of measurement property**	**Definition**
			PRO instrument are an adequate reflection of the performance of the items of the original version of the HR-PRO instrument
	Criterion validity		The degree to which the scores of an HR-PRO instrument are an adequate reflection of a ‘‘gold standard’’
**Responsiveness**			The ability of an HR-PRO instrument to detect change over time in the construct to be measured
	Responsiveness		Idem responsiveness
**Interpretability**[b]			The degree to which one can assign qualitative meaning - that is, clinical or commonly understood connotations - to an instrument’s quantitative scores or change in scores.

Abbreviations: HR-PROs, health-related patient-reported outcomes; CTT, classical test theory.

a The word ‘‘true’’ must be seen in the context of the CTT, which states that any observation is composed of two components - a true score and error associated with the observation. ‘‘True’’ is the average score that would be obtained if the scale were given an infinite number of times. It refers only to the consistency of the score and not to its accuracy (ref Streiner & Norman [12]).

b Interpretability is not considered a measurement property but an important characteristic of a measurement instrument.
(Reproduced with permission from Caroline Terwee, COSMIN)

For peer review only

Bibliography

1. Levey AS, Coresh J. Chronic kidney disease. Lancet (London, England) 2012;**379**(9811):165-80.
2. K/DOQI clinical practice guidelines for chronic kidney disease: evaluation, classification, and stratification. American journal of kidney diseases : the official journal of the National Kidney Foundation 2002;**39**(2 Suppl 1):S1-266.
3. Meyer T, Hostetter, T. Medical Progress: Uremia. The New England journal of medicine 2007;**13**(357):1316-25.
4. Almutary H, Bonner A, Douglas C. Symptom burden in chronic kidney disease: a review of recent literature. Journal of Renal Care 2013;**39**(3):140-50.
5. Gapstur RL. Symptom burden: a concept analysis and implications for oncology nurses. Oncology nursing forum 2007;**34**(3):673-80.
6. Gansevoort RT, Matsushita K, van der Velde M, et al. Lower estimated GFR and higher albuminuria are associated with adverse kidney outcomes. A collaborative meta-analysis of general and high-risk population cohorts. Kidney international 2011;**80**(1):93-104.
7. Stringer S, Sharma P, Dutton M, et al. The natural history of, and risk factors for, progressive chronic kidney disease (CKD): the Renal Impairment in Secondary care (RIISC) study; rationale and protocol. BMC nephrology 2013;**14**:95.
8. FDA. Patient-reported outcome measures: use in medicinal product development to support labeling claims. Guidance for industry 2009. https://www.fda.gov/downloads/drugs/guidances/ucm193282.pdf (accessed Feb 2017).
9. Go AS, Chertow GM, Fan D, et al. Chronic Kidney Disease and the Risks of Death, Cardiovascular Events, and Hospitalization. New England Journal of Medicine 2004;**351**(13):1296-305.
10. Basch E, Bennett A, Pietanza MC. Use of patient-reported outcomes to improve the predictive accuracy of clinician-reported adverse events. Journal of the National Cancer Institute 2011;**103**(24):1808-10.
11. Bren L. ‘The importance of patient-reported outcomes…It’s all about the patients’. FDA Consumer 2006. https://permanent.access.gpo.gov/lps1609/www.fda.gov/fdac/features/2006/606_patients.html (accessed Feb 2017).
12. Hjollund NH, Larsen LP, Biering K, et al. Use of Patient-Reported Outcome (PRO) Measures at Group and Patient Levels: Experiences From the Generic Integrated PRO System, WestChronic. Interactive journal of medical research 2014;**3**(1):e5.
13. Velikova G, Booth L, Smith AB, et al. Measuring quality of life in routine oncology practice improves communication and patient well-being: a randomized controlled trial. Journal of clinical oncology : official journal of the American Society of Clinical Oncology 2004;**22**(4):714-24.
14. Gilbody SM, Whitty PM, Grimshaw JM, et al. Improving the detection and management of depression in primary care. Quality & safety in health care 2003;**12**(2):149-55.
15. Bennett AV, Jensen RE, Basch E. Electronic patient-reported outcome systems in oncology clinical practice. CA: a cancer journal for clinicians 2012;**62**(5):337-47.
16. McCann L, Maguire R, Miller M, et al. Patients' perceptions and experiences of using a mobile phone-based advanced symptom management system (ASyMS) to monitor and manage chemotherapy related toxicity. Eur J Cancer Care (Engl) 2009;**18**(2):156-64.
17. myhealth@QEHB University Hospitals Birmingham NHS Foundation Trust 2016. http://www.uhb.nhs.uk/myhealth-at-QEHB.htm (accessed Feb 2017).
18. EHI 2014 Awards. Digital Health 2014. http://www.ehilive.co.uk/ehi-awards/past-winners/2014-awards/best-use-of-technology-to-share-information-with-patients-and-carers/ (accessed Feb 2017) (accessed Feb 2017).
19. Usability.Gov. https://www.usability.gov/ (accessed Feb 2017).

For peer review only

20. Nielsen J. Usability Engineering. Academic Press Inc 1994:165.
21. Tangri N, Stevens LA, Griffith J, et al. A predictive model for progression of chronic kidney disease to kidney failure. Jama 2011;**305**(15):1553-9.
22. Van Den Haak M, De Jong M, Jan Schellens P. Retrospective vs. concurrent think-aloud protocols: testing the usability of an online library catalogue. Behaviour & information technology 2003;**22**(5):339-51.
23. Terwee CB, Mokkink LB, Knol DL, et al. Rating the methodological quality in systematic reviews of studies on measurement properties: a scoring system for the COSMIN checklist. Quality of life research : an international journal of quality of life aspects of treatment, care and rehabilitation 2012;**21**(4):651-7.
24. Braun V, Clarke V. Using thematic analysis in psychology. Qualitative research in psychology 2006;**3**(2):77-101.
25. Bryman A. Social Research Methods. Oxford: Oxford University Press 2001.
26. Fusch Patricia NL. Are We There Yet? Data Saturation in Qualitative Research. The Qualitative Report 2015;**20**(9).
27. Terwee CB. Protocol for systematic reviews of measurement properties. COSMIN 2011. http://www.cosmin.nl/images/upload/files/Protocol%20klinimetrische%20review%20version%20nov%202011.pdf (accessed Feb 2017).
28. INVOLVE. Payment for Involvement - A guide for making payments to members of the public actively involved in NHS, public health and social care research. NIHR 2010. http://www.invo.org.uk/wp-content/uploads/2012/11/INVOLVEPayment-Guiderev2012.pdf (accessed Feb 2017).
29. Draper H, Wilson S, Flanagan S, et al. Offering payments, reimbursement and incentives to patients and family doctors to encourage participation in research. Family practice 2009;**26**(3):231-38.
30. Parliament of the United Kingdom of Great Britain and Northern Ireland. Data Protection Act 1998. 2005:92. http://www.legislation.gov.uk/ukpga/1998/29/pdfs/ukpga_19980029_en.pdf (accessed Feb 2017).
31. DOH. Confidentiality - NHS Code of Practice. Department of Health 2003. https://www.gov.uk/government/publications/confidentiality-nhs-code-of-practice (accessed Feb 2017).
32. DOH. The Caldicott Committee. Report on the Review of Patient-Identifiable Information Department of Health 1997. http://webarchive.nationalarchives.gov.uk/+/www.dh.gov.uk/en/Publicationsandstatistics/Publications/PublicationspolicyandGuidance/DH_4068403 (accessed Feb 2017).
33. UoB. Code of practice for research. University of Birmingham 2015 - 2016. http://www.birmingham.ac.uk/Documents/university/legal/research.pdf (accessed Feb 2017).
34. UoB. Guidance on out of hours activities and lone working. University of Birmingham 2012. https://intranet.birmingham.ac.uk/hr/documents/public/hsu/hsuguidance/31ohalw.pdf (accessed Feb 2017).

For peer review only

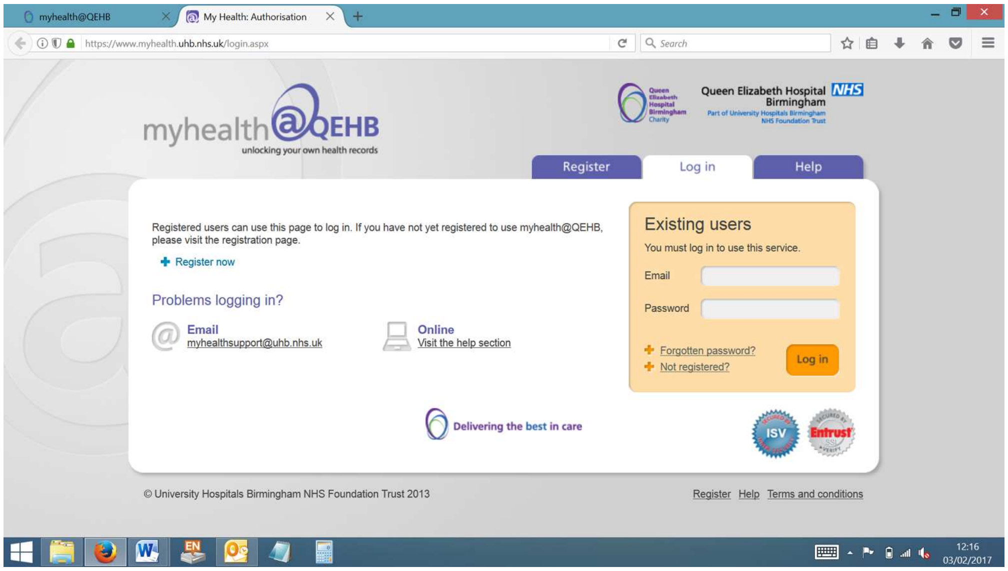

Fig 1. Screenshot of the myhealth@QEHB login page

677x381mm (72 x 72 DPI)

review only

Using Patient-Reported Outcome measures (PROMs) to promote quality of care and safety in the management of patients with Advanced Chronic Kidney Disease (PRO-trACK Project) – A mixed-methods project protocol

Journal:	*BMJ Open*
Manuscript ID	bmjopen-2017-016687.R1
Article Type:	Protocol
Date Submitted by the Author:	16-May-2017
Complete List of Authors:	Aiyegbusi, Olalekan; University of Birmingham, Institute of Applied Health Research; University of Birmingham, Centre for Patient-Reported Outcomes Research (CPROR)<br>Kyte, Derek; University of Birmingham, Institute of Applied Health Research; University of Birmingham, Centre for Patient-Reported Outcomes Research (CPROR)<br>Cockwell, Paul; Queen Elizabeth Hospital Birmingham, Department of Renal Medicine; University of Birmingham, Centre for Patient-Reported Outcomes Research (CPROR)<br>Marshall, Tom; University of Birmingham, Institute of Applied Health Research; University of Birmingham, Centre for Patient-Reported Outcomes Research (CPROR)<br>Dutton, Mary; Queen Elizabeth Hospital Birmingham, Department of Renal Medicine<br>Slade, Anita; University of Birmingham, Institute of Applied Health Research; University of Birmingham, Centre for Patient-Reported Outcomes Research (CPROR)<br>Marklew, Neil; University of Birmingham, Patient Advisory Group Member, Centre for Patient-Reported Outcomes Research (CPROR)<br>Price, Gary; University of Birmingham, Patient Advisory Group Member, Centre for Patient-Reported Outcomes Research (CPROR)<br>Verdi, Rav; University of Birmingham, Patient Advisory Group Member, Centre for Patient-Reported Outcomes Research (CPROR)<br>Waters, Judi; University of Birmingham, Patient Advisory Group Member, Centre for Patient-Reported Outcomes Research (CPROR)<br>Sharpe, Keeley; University of Birmingham, Patient Advisory Group Member, Centre for Patient-Reported Outcomes Research (CPROR)<br>Calvert, Melanie; University of Birmingham, Institute of Applied Health Research; University of Birmingham, Centre for Patient-Reported Outcomes Research (CPROR)
**Primary Subject Heading**:	Patient-centred medicine
Secondary Subject Heading:	Health services research, Qualitative research, Renal medicine, Research methods
Keywords:	NEPHROLOGY, Chronic renal failure < NEPHROLOGY, QUALITATIVE RESEARCH, STATISTICS & RESEARCH METHODS, USABILITY TESTING,

For peer review only - http://bmjopen.bmj.com/site/about/guidelines.xhtml

	VALIDATION STUDY

SCHOLARONE™ Manuscripts

For peer review only

Using Patient-Reported Outcome measures (PROMs) to promote quality of care and safety in the management of patients with Advanced Chronic Kidney Disease (PRO-trACK Project) – A mixed-methods project protocol

Olalekan Lee Aiyegbusi[1, 2], Derek Kyte[1, 2*], Paul Cockwell[1, 3], Tom Marshall[1, 2], Mary Dutton[3], Anita Slade[1, 2], Neil Marklew[4], Gary Price[4], Rav Verdi[4], Judi Waters[4], Keeley Sharpe[4], Melanie Calvert[1, 2]

1. Centre for Patient-Reported Outcomes Research, University of Birmingham, Edgbaston, Birmingham, UK
2. Institute of Applied Health Research, University of Birmingham, Edgbaston, Birmingham, UK
3. Department of Renal Medicine, University Hospitals Birmingham NHS Foundation Trust, Queen Elizabeth Hospital Birmingham, Mindelsohn Way, Edgbaston, Birmingham, UK
4. Patient Advisory Group Member, Centre for Patient-Reported Outcomes Research, University of Birmingham, UK

Email: Dr O.L. Aiyegbusi, oxa238@bham.ac.uk - Dr D. Kyte, d.g.kyte@bham.ac.uk - Prof P. Cockwell, paul.cockwell@uhb.nhs.uk - Prof T. Marshall, t.p.marshall@bham.ac.uk - M. Dutton, Mary.Dutton@uhb.nhs.uk - Dr A. Slade, A.L.Slade@bham.ac.uk - Prof M. Calvert, m.calvert@bham.ac.uk

* **Correspondence**: Derek Kyte, d.g.kyte@bham.ac.uk

Word count: 6,192

For peer review only - http://bmjopen.bmj.com/site/about/guidelines.xhtml

ABSTRACT

Introduction: Advanced chronic kidney disease (CKD) has a major effect on the quality of life and health status of patients and requires accurate and responsive management. The use of electronic patient reported outcome measures (ePROMs) could assist patients with advanced pre-dialysis CKD, and the clinicians responsible for their care, by identifying important changes in symptom burden in real time. We report the protocol for 'Using Patient-Reported Outcome measures (PROMs) to promote quality of care and safety in the management of patients with Advanced Chronic Kidney Disease' (PRO-trACK) project, which will explore the feasibility and validity of an ePROM system for use in patients with advanced CKD.

Methods and analysis: The project will utilise a mixed-methods approach in three studies: (i) usability testing of the ePROM system involving up to 30 patients and focusing on acceptability and technical performance/stability; (ii) ascertaining the views of patient and clinician stakeholders on the optimal use and administration of the CKD ePROM system - this will involve qualitative face-to-face/telephone interviewing with up to 30 patients or until saturation is achieved; focus groups with up to 15 clinical staff, management and IT team members; (iii) psychometric assessment of the system, within a cohort of at least 180 patients with advanced CKD, to establish the measurement properties of the ePROM.

Ethics and dissemination: This project was approved by the West Midlands Edgbaston Research Ethics Committee (Reference 17/WM/0010) and received HRA Approval on 24/02/2017.

The findings from this project will be provided to clinicians at the Department of Renal Medicine, Queen Elizabeth Hospitals, Birmingham (QEHB), NHS England,

For peer review only - http://bmjopen.bmj.com/site/about/guidelines.xhtml

presented at conferences and to the Kidney Patients' Association, British Kidney Patient Association and the British Renal Society. Articles based on the findings will be written and submitted for publication in peer-reviewed journals.

Strengths and limitations of this project

- Whilst there is evidence to support the use of ePROMs in the management of other conditions, notably cancer, the evidence for the use of ePROMs in the management of patients with CKD is currently limited. The PRO-trACK project will help fill this evidence gap.
- By using a mixed methods approach, the project will provide a rigorous exploration of the acceptability, validity and feasibility of the ePROM system for the management of patients with CKD.
- This project will only involve patients with CKD stages 4 and 5 and patients on dialysis for $\leq$ 6 months. This is because the ePROM system is presently intended for patients with advanced CKD stages 4 and 5 who we hypothesise are likely to derive the most benefit.

For peer review only

For peer review only - http://bmjopen.bmj.com/site/about/guidelines.xhtml

INTRODUCTION

Chronic kidney disease (CKD) refers to a number of disorders affecting the structure and function of kidneys.[1] The definition of CKD is based on sustained reduction in renal function (i.e. "estimated glomerular filtration rate (eGFR) <60 mL/min per 1·73 m² for 3 months or more") and/or evidence of structural or functional abnormalities of the kidneys regardless of clinical diagnosis.[1 2] CKD is associated with other long-term conditions such as hypertension, cardiovascular diseases and diabetes will increase the risk of ill health both when present singly and when associated with other long-term conditions.

CKD causes clinical signs and symptoms, particularly when the disease is advanced.[3] The most commonly experienced are fatigue, drowsiness, pain, pruritus and dry skin.[4] These symptoms often occur concurrently and may negatively affect patients' daily activities and their physical, emotional and psychological well-being.[5] Therefore impacting on the quality of life of (QOL) of those affected, particularly as the disease progresses towards end-stage renal disease.[6 7]

The symptoms of CKD progression can be monitored using self-completed questionnaires known as patient-reported outcome measures (PROMs) which capture information about health status from patients' own point of view.[8] Although commonly administered in paper format, PROMs can be completed as electronic patient-reported outcome measures (ePROMs) using multiple digital platforms. This makes it possible to remotely monitor patients and generate 'real time' data about patient symptoms and QOL. As patients with advanced CKD are at risk of deteriorating rapidly and developing cardiovascular complications,[9] the use of an ePROM system may help clinicians detect deterioration of symptoms and assist with

For peer review only - http://bmjopen.bmj.com/site/about/guidelines.xhtml

the tailoring of treatment to the needs of each patient.[10-12] Health-related issues that matter to patients may also be identified using ePROM data and this could potentially facilitate communication and shared-decision making between patients and their clinicians.[13-15] In stable patients, the use of ePROMs may reduce the occurrence of unnecessary clinical appointments.[12]

In Denmark the WestChronic ePROM System has been successfully implemented for tailoring the care of various patient groups[12], while in the UK, patients with cancer have been successfully monitored for the side effects of chemotherapy using the ePROM Advanced Symptom Management System (ASyMS).[16] However, there is limited information on the use of ePROMs in the management of adult patients with CKD in a routine clinical setting. Therefore, the aim of the project is to explore the feasibility and validity of an electronic patient-reported outcome measure (ePROM) system for monitoring and assisting with the individual management of patients with advanced CKD.

Questionnaire selection and the ePROM system

Selection of measures was informed by i) a systematic review of measurement properties of PROMs used in CKD patients[17] and ii) feedback from the PAG. The systematic review found evidence to support the use of the KDQOL-SF and KDQOL-36. However, these two measures were validated by very few studies in our population of interest (stages 4 and 5 CKD). The review also identified the IPOS-Renal, which is currently undergoing validation through use in a number of renal units in the UK.

For peer review only - http://bmjopen.bmj.com/site/about/guidelines.xhtml

A patient advisory group (PAG) met prior to commencing the project and considered the acceptability, burdensomeness and relevance of the KDQQOL-SF, KDQOL-36 and the IPOS-Renal for the target CKD group. The PAG members chose the KDQOL-36 and IPOS-Renal as they were brief and easy to understand. Therefore, the decision was made to validate the electronic versions of these in the pre-dialysis population (stage 4 and 5). The EuroQol 5-dimension 5-Level (EQ-5D-5L) questionnaire will be used as a comparison measure for this validation study.

The KDQOL-36 and the IPOS-Renal are free to use without charge as long as the developers are appropriately acknowledged and cited. The EQ-5D requires prior written consent and payment of licensing fees (if applicable). A license will be obtained for this project.

The PRO-trACK project will consist of 3 studies namely: (i) usability testing with patients (ii) qualitative study with patients and clinicians (iii) validation study with patients.

Whilst the usability testing and qualitative interviews are related, they are distinct studies. The usability testing will focus on the actual experience of patients as they test the ePROM system while the qualitative interviews will explore the broader opinions of patients on the use of ePROMs in the NHS.

The content validation of the KDQOL-36 and the IPOS-Renal by patients and clinicians during the qualitative study as well as the results of the validation study will assist the research team with the final decision on which measure to take forward for the final version of the ePROM system.

The ePROM system will be designed as an electronic method of allowing patients with CKD to remotely self-report their symptoms and quality of life using a digital

For peer review only - http://bmjopen.bmj.com/site/about/guidelines.xhtml

platform that is convenient to them (PC, tablet, smartphone, telephone voice recognition or scanned paper copy), providing important patient-centred data to the patients' clinical team.

The ePROM system will be accessed via the secure electronic patient portal developed by the University Hospitals Birmingham NHS Foundation Trust, known as 'myhealth@QEHB' (See Figure 1).[18] myhealth@QEHB currently has 14,000 patient users and was awarded the prestigious E- Health Insider award in 2014.[19] Around 1200 renal patients are currently signed up for myHealth@QEHB. At the moment this is a voluntary system.

Table 1. Description of questionnaires

Measure	Description
Kidney Disease Quality of Life - 36 (KDQOL™-36)	A 36-item HRQOL measure designed for use in kidney disease patients undergoing dialysis. Derived from the KDQOL-SF.[20]<br>There are 3 disease-specific dimensions namely: (i) symptoms and problems (6 items) (ii) burden of kidney disease (4 items) (iii) effects of kidney disease (8 items).It also includes two summary scales derived from the generic SF-12 namely: (i) the physical component summary (PCS – 6 items) (ii) mental component summary (MCS – 6 items).[21]<br>Response options vary for the items and range from 2 to 6. Questions 1, 8, 12 – 36 have five response options; questions 2 and 3 have three response options: questions 4 - 7 have two response options; and questions 9 – 11 have six response options each. Total and subscale scores may be calculated using the KDQOL-36™ scoring program. The raw scores for each item is converted linearly to a 0 to 100 range with higher scores indicating better HRQOL.[20]
Integrated Patient	A symptom-specific measure with 11 questions for use with patients with

For peer review only - http://bmjopen.bmj.com/site/about/guidelines.xhtml

Table 1. Description of questionnaires	
**Measure**	**Description**
Outcome Scale (IPOS)-Renal	advanced kidney disease to assess their care needs. The questions relate to common symptoms renal patients experience plus additional items such as information needs, practical issues, family anxiety.[22] The first question has a free text response format. Questions 2 to 9 have five response options while questions 10 and 11 have three response options each. The measure is currently being validated by researchers at the Department of Palliative Care, Policy and Rehabilitation at King's College London. Dimensions yet to be ascertained.
EuroQol EQ-5D-5L	A generic utility measure with a self-classifier and a visual analogue scale (VAS) which can be used to measure health status.[23 24] The self-classifier includes 5 dimensions: (i) mobility (ii) self-care (iii) usual activities (iv) pain/discomfort (v) anxiety/depression This version of the measure has 5 levels of severity (response options) for each dimension. It is possible to describe 3,125 different health states between 0 (dead) and 1 (perfect health).[23 24]

For peer review only - http://bmjopen.bmj.com/site/about/guidelines.xhtml

METHODS AND ANALYSIS

Project Design

In this section, the project setting and eligibility criteria for participants will be described first as this will be the same for the three studies. Aspects of research methods specific to each study will be subsequently discussed separately.

Project Setting

Queen Elizabeth Hospitals, Birmingham (QEHB) will be the host site for this project. Clinical staff at the Renal Unit, QEHB and academic researchers at the Centre for Patient Reported Outcomes Research, University of Birmingham will be responsible for the conduct and management of the project. The nephrology service comprises 21 consultants, 30 junior doctors, and 20 nurses and allied health professionals in the CKD team.

Project Participants

Patient participants

For the project, we will recruit adult patients with advanced CKD stages 4 and 5 under the care of the renal services at QEHB, with eGFR $\leq$ 20ml/min/1.73m^2 who have been counselled about treatment modalities for end-stage renal disease (ESRD). In addition, using the renal risk calculator, they must have a >20% projected risk of progressing to ESRD and requiring renal replacement therapy (RRT) or an eGFR <10ml/min/1.73m^2 within 2-years.[25] The renal risk calculator is a model

For peer review only - http://bmjopen.bmj.com/site/about/guidelines.xhtml

designed to use routinely collected laboratory tests to predict the progression of patients with CKD stages 3 to 5 to kidney failure.[25]

We have selected this group of patients as our main target for this project because, even though they have not yet reached ESRD, they are likely to have high symptom burden and a high risk of rapid clinical deterioration to renal failure. We hypothesise this group of patients are likely to derive the most benefit from an ePROM system. The research team also is working on a related project focused on dialysis patients. Patients who have commenced dialysis within 6 months will also be eligible to participate as we hypothesise they will be able to recall their symptoms and medical needs pre-dialysis. Participants will be required to converse in everyday English and provide informed consent.

Patients who have a recent history of acute kidney injury within the last 3 months, a co-morbidity with a high level of symptoms or terminal illness likely to lead to the death within 6 months of participation will be excluded from the study.

We will aim to recruit different sets of eligible patients for each study in order to minimise participant burden. However, if patients voluntarily express an interest, they will be allowed to participate in more than one study as long as the renal team is satisfied with their health status.

Efforts will be made to recruit up to 30% of the study participants from minority ethnic groups to reflect the ethnic diversity of the patient catchment area.

Although our previous work does not show an influence of socio-economic status (SES) on outcomes for CKD,[26] we will be mindful of sample diversity in relation to socio-demographic variables such age, gender, ethnicity and other relevant socio-demographic factors. We will collect data on participant characteristics to monitor this as recruitment and qualitative data collection progress. Although we appreciate

For peer review only - http://bmjopen.bmj.com/site/about/guidelines.xhtml

that patient populations and research samples do not always represent such diversity, we will try to employ recruitment strategies that optimise our ability to recruit a diverse patient sample.

Clinicians and other professional staff

Clinicians who manage patients with CKD at the Renal Unit, QEHB and members of the myHealth team and hospital management staff who provide consent will be recruited for this project.

Recruitment methods

A member of the renal research team at QEHB will screen patients for eligibility using the electronic screening tools that are utilised for clinical purposes. This will identify patients who meet the eligibility criteria and the clinics they attend. Eligibility will be confirmed by direct review of the clinical records by the research nurse and a clinician who are members of the renal care team, on the delegated duty log. Patients will then be approached at clinic by a member of the renal care team and given patient information sheet to read. Further information about the study will be given and their immediate queries will be addressed. They will be contacted no earlier than 48 hours after the clinic visit to ascertain if they wish to participate in the study.

Clinicians and other professional staff will be recruited by the members of the research team and sent information sheets via email.

For peer review only - http://bmjopen.bmj.com/site/about/guidelines.xhtml

Study 1 – Usability testing

Usability testing will be conducted to evaluate the acceptability of the ePROM system.

Study objectives

i. To determine whether patients can easily navigate the ePROM system.

ii. To determine if patients are able to complete the questionnaires successfully on their own, and if not, how much assistance they require.

iii. To determine the average length of time required to complete an ePROM report.

iv. To determine the level of satisfaction with the ePROM interface.

v. To identify changes that might be required to improve user performance and satisfaction.

Data collection

Usability testing refers to the appraisal of a product or service by potential service users and involves the observation of such users completing a task within a predetermined scenario.[27 28]

The scenario for this study will be the self-report of a patient's health status between clinic appointments using an electronic device such as a smartphone, tablet or PC/laptop. Each patient will undergo a single one-to-one session with (OLA) the project's Chief Investigator (CI), and attempt to complete the 3 electronic questionnaires with as little assistance as possible.

For peer review only - http://bmjopen.bmj.com/site/about/guidelines.xhtml

The Concurrent Think Aloud (CTA) and Retrospective Probing (RP) moderating techniques will be used for this study.[29] CTA involves the thinking aloud and vocalisation of participants' thoughts during the session while RP refers to interviewing the participant following the completion of the session.[29] The advantage of combining both techniques is that real time feedback could be obtained for exploration by the CI afterwards.[27]

The CI will take detailed notes of the participants' comments, actions, non-verbal cues and errors and pass minimal comments to encourage them to think aloud during the sessions.

Qualitative data will be collected in form of a brief audio-recorded interview at the end of each test. The patients will be questioned based on the notes taken by the CI during the session. They will be encouraged to provide any recommendation to improve user experience.

Data analysis

Thematic analysis will be conducted on the qualitative data (See the analysis of patient interviews for more details), which will include the notes taken during the sessions and the transcripts of the post-test interview.

Quantitative data will be summarised using descriptive statistics such as proportions, averages, percentages and rates.[27] Quantitative data will include successful completion rates, error-free rates and average time required for completion.

For peer review only - http://bmjopen.bmj.com/site/about/guidelines.xhtml

Sample size

We will recruit up to 30 patients from QEHB for this study based on the recommendations found in literature.[30] The process of improving the usability of any system is an iterative one;[27] therefore a minimum of 2 testing cycles will be conducted with the patients. The findings from the first test cycle will guide the process of improving the ePROM system before the second cycle is conducted.

For peer review only

For peer review only - http://bmjopen.bmj.com/site/about/guidelines.xhtml

Study 2 – Qualitative Study

This study will explore the views of stakeholders on the optimal use and administration of the CKD ePROM system.

Study 2a - Patient interviews

Study objectives

i. To determine which symptoms patients with CKD find most bothersome.
ii. To explore how acceptable ePROMs are.
iii. To determine how often patients will be willing to complete the ePROM and their preferred method of completion i.e., PC, smartphone, tablet, telephone voice recognition or paper completion.
iv. To explore the likely factors that may improve or discourage the completion of ePROMs.
v. To explore how they would like to receive feedback from the clinical team regarding the ePROMs they provide.

Data collection

Semi-structured face-to-face interviews will be arranged to either coincide with patients' scheduled clinic visit or held on a separate day if preferred. The option of a telephone interview will also be given.

A topic guide will be used to provide a general direction for each interview and ensure that important issues are covered whilst allowing enough flexibility to capture other relevant themes that may be arise during any session.

For peer review only - http://bmjopen.bmj.com/site/about/guidelines.xhtml

Interviews will be recorded using an encrypted digital audio recorder and transcribed by a professional transcription company.

Data analysis

The transcripts will be analysed by the CI using the Nvivo 10 software package by QSR International. Thematic analysis of the data will be conducted following the six steps described by Braun and Clarke.[31] The process of analysis will begin with the CI 'actively' reading and engaging with the data set (i.e. searching for patterns and meanings). The next phase will be the initial coding of the raw transcript data using the QSR software. Extracts will be coded inclusively (i.e. a little surrounding data will be kept to retain contextual meaning).[32]

Phase 3 will involve the analysis and organisation of codes into potential themes. These initial themes will be revised and refined in the 4th phase on two levels. The first is at the level of coded data extracts to ensure they are coherent for each theme. The second level of analysis is to ensure the themes reflect the data set. During this phase redundant codes and themes may be removed, revised or merged as required. Phase 5 will involve the definition of what each theme is and what it is not; and its importance in relation to the entire data and the research questions. The themes will be considered individually as well as in relation to other themes to ensure overlaps are kept to a minimum. The final phase will be the production of the study report.

The project team (Independent of the CI) will randomly review a sample of transcripts for verification purposes.

For peer review only - http://bmjopen.bmj.com/site/about/guidelines.xhtml

Data analysis will be carried out simultaneously with data collection and both will continue until no new themes emerge from the further analysis i.e. data saturation has been reached.[33]

Respondent validation will be undertaken, whereby a summary of the main points arising from the interview will be sent to each participant for comments.

Sample size

Based on experience from previous similar qualitative studies conducted by the research team, recruitment will continue until a target sample size of between 15 and 30 patient participants is attained or until saturation is achieved.

For peer review only

For peer review only - http://bmjopen.bmj.com/site/about/guidelines.xhtml

Study 2b - Focus groups with clinicians and other professional staff

Focus group discussions will be held with clinicians who manage patients with CKD at the Renal Unit, QEHB, members of the myHealth team and hospital management staff as required.

Study objectives

i. To evaluate and rate the relevance of the items of the ePROM questionnaires with clinical staff (content validation).
ii. To determine those factors that may improve or discourage the use of ePROM data by clinicians.
iii. To determine clinicians' preferred method of displaying ePROM data.

Data collection

An independent member of staff will serve as chief moderator and direct the discussions while the CI will act as assistant moderator, making notes and observing the interactions within the groups. The discussions will be allowed to develop with minimum interference following a topic guide to ensure that all the main points are covered. Focus group sessions will be recorded using an encrypted digital audio recorder and transcribed by a professional transcription company.

Face-to-face or telephone interviews with clinicians and other professional staff may be conducted to explore their views on the use of PROMs. These interviews will require a maximum of 1 hour.

For peer review only - http://bmjopen.bmj.com/site/about/guidelines.xhtml

Sample size

We will aim to include up to 15 participants in up to 2 focus groups (7 to 8 participants in each group) and if necessary interview the same number of participants.

Data analysis

Thematic analysis will be conducted (See analysis of patient interviews for details).

For peer review only - http://bmjopen.bmj.com/site/about/guidelines.xhtml

For peer review only

Study 3 – ePROM Validation

The purpose of this study is to evaluate the measurement properties of the electronic versions of the KDQOL-36 and the IPOS-Renal against the EQ-5D-5L and clinical data. At the end of this study, the most suitable questionnaire(s) would be taken forward for formal feasibility testing.

Study objectives

i. To determine the reliability and validity of the ePROM questionnaires.
ii. To determine the ability of the ePROM questionnaires to detect change in a patient's health over a period of time.
iii. To determine which of the two questionnaires is most suitable to take forward for formal feasibility testing.

Data collection

Patients will be registered on 'myhealth@QEHB' in order to access the ePROM system. There will be the option of completing paper versions if preferred. Time for questionnaire completion may be influenced by patients' symptoms but should require no more than 1 hour.

All participants will be asked to complete the 2 questionnaires 3 times: at study entry, at 2 weeks after initial completion and at 6 months after initial completion. Completing the questionnaires at these time points will facilitate the comprehensive assessment of psychometric properties.[8]

For peer review only - http://bmjopen.bmj.com/site/about/guidelines.xhtml

Data analysis

Quantitative data will be analysed using statistical software such as STATA. Where appropriate, analysis will be conducted separately for patients with CKD stages 4 and 5 and patients on dialysis.

A disclaimer statement will be included in the patient information sheets (PIS) for the validation study informing the patients that the questionnaires will not be assessed until the end of the study; therefore, patients should inform their clinician (e.g. general practice or renal services) of any health care needs for management.

For peer review only

For peer review only - http://bmjopen.bmj.com/site/about/guidelines.xhtml

Psychometric evaluation

Classical test theory (CTT) and the Rasch measurement model[34] will be used to evaluate the psychometric properties of the ePROM versions of the KDQOL-36 and IPOS-Renal. CTT is a traditional approach to questionnaire development which postulates that a person's observed score consists of their true score plus an additional measurement error score, the underlying assumption is that this relationship is additive.[35 36] This additive model involves the summation of item ratings on a Likert-type scale to obtain a total score, however the values of the true score and error score cannot be determined and CTT does not describe the hierarchy of the items.[35-37]

Rasch analysis[34] is one method of evaluating measurement tools to ensure they deliver reliable and valid measurement and is increasingly being used in clinical research and practice for refinement and development of PRO.[38] The Rasch model operationalises axioms of additive conjoint measurement, and tests the extent to which PRO are uni-dimensional.[39] Fit to the Rasch model establishes that these axioms have been fulfilled through a number of fit statistics.[40 41] Rasch analysis is an iterative process identifying and studying anomalies in the data and the extent to which data conforms to the Rasch model. The degree of fit achieved will identify the extent to which KDQOL-36 and IPOS-Renal demonstrate construct validity, unidimensionality and reliability.[42] When data fit the Rasch model it confirms that the PRO are unidimensional summation of scores from the KDQOL-36 and IPOS-Renal is legitimate.[40]

CTT will be used to evaluate the reliability, construct validity and responsiveness while Rasch analysis will be done to complement the CTT assessment of structural validity of the two questionnaires.

For peer review only - http://bmjopen.bmj.com/site/about/guidelines.xhtml

For peer review only

For definition of measurement properties see Table 2.

Classical test theory (CTT) methods

Factor analysis

i) Structural validity: Exploratory factor analysis will be used to evaluate the factor structure of the KDQOL-36 and the IPOS-Renal.[43 44] This will be conducted using principal component analysis (PCA) with orthogonal Varimax rotation of quadrants.[43] Factors will be identified based on the Scree test and the percentage of variance accounted for by a particular factor.[45 46] Eigenvalues measure the amount of variation and factors will be required to have a minimum eigenvalue of 1.0.[45] Subsequently, a confirmatory factor analysis will be conducted to test whether the hypothesized factor models of the questionnaires are supported by actual data.[43 47]

Reliability

i) *Internal consistency:* Using baseline scores, Cronbach's alpha[48] will be calculated for the total scale and subscale scores of the KDQOL-36 and IPOS-Renal. Alpha values 0.70 – 0.90 will be deemed acceptable[49-51] An 'if item deleted' analysis will be conducted to identify whether any items should be dropped from the scale.[52]

ii) *Test–retest reliability:* The completion of the electronic questionnaires, 2 weeks after the initial completion will allow the assessment of the stability

For peer review only - http://bmjopen.bmj.com/site/about/guidelines.xhtml

of the questionnaires.[53] Intra-class correlation coefficients (ICC) for agreement will be calculated on subscale and total scores using a two-way random effects model.[54 55] ICC values > 0.75 will indicate excellent test-retest reliability, values 0.40 - 0.75 will be considered good, while values < 0.4 will indicate weak agreement.[35 53 56]

iii) *Measurement error:* As we are not aware of values for measurement error and minimally important clinical change (MIC) for our population of interest, these will be calculated in this study. Measurement error will be calculated using the standard error of measurement (SEM).[54 57] The MIC will be determined for patients that commence dialysis within the study period using a patient-reported anchor based method. This is regarded as the ideal method for calculating the MID as it captures the patients' values directly.[58] We will compare changes in measurement scores with a patient-reported global rating of change scale as our reference 'anchor'.[58-60] The measurement error and the MIC will assist with the assessment and interpretation of treatment outcomes and effects.[61]

Construct validity

ii) Convergent validity (Hypothesis testing): We have formulated the following hypotheses to test in order to establish convergent validity. Pearson or Spearman's correlation coefficients will be calculated for correlations as appropriate. Pearson's or Spearman's correlation coefficients $\geq$ 0.40 will be considered acceptable for scales that are theoretically related.[62 63]

For peer review only - http://bmjopen.bmj.com/site/about/guidelines.xhtml

Hypothesis 1 – Each item of the KDQOL-36 and the IPOS-Renal will have a positive correlation $\geq$ 0.40 with its own hypothesised subscale after correction for overlap.[64] IPOS-Renal items will be correlated with their subscales once these have been established by factor analysis.

Hypothesis 2 – The generic (SF-12) and the disease-specific domains of the KDQOL-36 will have weak to moderate positive correlations with each other as they are designed to assess different aspects of health-related quality of life.[65 66]

Hypothesis 3 – Each subscale score of the KDQOL-36 will have positive correlations with the overall health rating score (question 1 of KDQOL-36).[67]

Hypothesis 4 – The generic (SF-12) subscales, the physical component summary (PCS) and the mental component summary (MCS) of the KDQOL-36 will have higher positive correlations with the utility scores of the EQ5D5L than the kidney-specific subscales of the KDQOL-36 and the symptom-specific scales of the IPOS-Renal.

Hypothesis 5 – Clinical parameters specific to kidney disease such as the eGFR, will correlate better with dialysis-targeted dimensions of the KDQOL-36 and the IPOS-Renal than with generic dimensions of KDQOL-36.[68]

Hypothesis 6 – The utility scores of the EuroQol VAS will have a high positive correlation with the overall health rating scores of the KDQOL-36.

Hypothesis 7 – The comparisons of the means of the lowest scoring 25 percentile and the higher scoring 75 percentile for each disease-specific

For peer review only

For peer review only - http://bmjopen.bmj.com/site/about/guidelines.xhtml

subscale of the KDQOL-36 will be statistically significant (P values < 0.05, using the Mann-Whitney U test).[69]

Responsiveness

The questionnaires will be administered 6 months after the initial completion in order to assess the ability of the questionnaires in detecting changes in patients' condition. Using Pearson's correlation coefficient, we will test three hypotheses for responsiveness based on changes in scores as recommended by the COSMIN group.[70]

Hypothesis 1 – There will be significant changes in the QOL scores of patients who switch from conservative management to RRT within this period. Therefore, the QOL scores before and after commencing RRT will be compared using the Wilcoxon signed-rank test.[71]

Hypothesis 2 – Changes in KDQOL-36 scores for patients who switch to RRT from conservative care will correlate negatively with changes in their creatinine values and correlate positively with changes in residual renal function and serum albumin.[68 70]

Hypothesis 3 – There will be positive correlations between the global rating scale and the changes in KDQOL-36 scores for patients who switch to RRT from conservative care.[70]

In addition to these hypotheses, effect sizes (ES) and standardized response mean (SRM) will be calculated for patients with CKD stages 4 & 5 who were initially managed conservatively but progressed to renal failure (on RRT) during the study period.[72 73]

For peer review only - http://bmjopen.bmj.com/site/about/guidelines.xhtml

Higher ES or SRM indicate greater responsiveness and values up to 0.2 will be regarded as small; 0.5 moderate and 0.8 as substantial according to Cohen's criteria.[74] Receiver operating characteristic (ROC) curves will be used to establish a cut point for predicting transition to RRT.

Application of Rasch analysis

The underlying assumption with the Rasch model is that individual items capture a single underlying trait, and therefore the summation of items from the KDQOL-36 or IPOS-Renal form unidimensional scales. Rasch analysis is an iterative process that identifies and studies anomalies in the data and the extent to which KDQOL-36 or IPOS-Renal data conforms to the Rasch model and therefore the extent to which the instrument is unidimensional. Fit will be established using a variety of indicators and fit statistics.[75] The Rasch Unidimensional Measurement Model software (RUMM2030)[76] will be used to analyse KDQOL-36 and IPOS-Renal data.

KDQOL-36 has a mixture of dichotomous and polytomous responses whereas the and IPOS-Renal items are all polytomous using a Likert response format (See Table 1). Affirmation of response categories by respondents should follow a logical sequence. As their perceived level of health improves/deteriorates, then responses should reflect this by affirming higher or lower scoring categories, moving from a score of 1, to 2, then 3 etc. on any item.[77] Rasch Andrich thresholds are the points between adjoining categories where the probability of affirming either category is 50/50, when responders' perceived level of health is captured by the adjoining categories is equidistant.[78] Where there is agreement with this expected hierarchy, response thresholds appear ordered, lack of conformity is observable as disordered

For peer review only - http://bmjopen.bmj.com/site/about/guidelines.xhtml

thresholds.[78] Disordered thresholds can be indicative of poorly defined or redundant scoring categories and consequently conceptual distinctions between categories maybe imprecise. Responders then find it difficult to assign a category to their perceived health status or QOL.

Targeting will be established by examining the extent to which distributions of participants perceived QOL/health status and levels of health identified by-KDQOL-36 and IPOS-Renal items are analogous. The position of each responder and item on the underlying construct is defined as the person's ability and the items difficulty.[36] Therefore, responders with low levels of perceived health or QOL should only affirm items and scoring categories which capture low levels of health. The item which captures the average level of competence will be identified as having zero logits by RUMM2030. Therefore, when person and items are appropriately targeted, person mean location scores should approximate the zero value of the item locations. A positive mean value for responders estimated locations will suggest that responders' average levels of competence are higher than the average KDQOL-36 and IPOS-Renal item location. Conversely negative person locations will confirm the opposite to be true.[40]

The person separation index (PSI) uses the logit values to estimate the internal reliability of the KDQOL-36 and IPOS-Renal and is conceptually equivalent to Cronbach's Alpha. It is an estimate of the proportion of the error free variance of the person estimates relative to the sum of the variance and the error variance in these estimates. It identifies the extent to which the instrument is able to discriminate between groups with different health states.[79] Minimum PSI value suggested for group use is 0.70 and for individual use 0.85.[40]

For peer review only - http://bmjopen.bmj.com/site/about/guidelines.xhtml
For peer review only

Individual tests of fit for each person and item will reflect the difference between responders observed and expected responses if data fits the Rasch model. RUMM2030 will automatically cluster responders into equivalent size groups (class intervals) according to their overall ability. A number of statistics utilise these class intervals including χ^2 statistics and residual values.[80] Residuals are summations of individual item or person deviations from expected fit to the Rasch model, standardised as a z-score. Residual scores between ± 2.5 will indicate adequate fit to the Rasch model.[80]

A χ^2 statistic will compare this difference, with a summed χ^2 for each class interval contributing to the overall χ^2 for that item. The χ^2 for all items will then be summed to demonstrate the overall item-interaction statistic. A non-significant χ^2 interaction statistic will indicate theoretical fit to the Rasch model.[80] A significant χ^2 will indicate the need for further evaluation to establish potential causes of misfit.

Differential item functioning (DIF) is a form of item bias that can affect fit to the model. DIF manifests itself as responders responses to individual items by sample sub-groups (e.g. gender or age group) inconsistent with their overall perceived level of health.[81] DIF will be identified using ANOVA and statistically significant probability (p<.05, or a Bonferroni corrected level). DIF for gender, age group and kidney disease stage will be examined.

Local independence of items is an underlying principle of the Rasch Model.[42] Response dependency occurs when the response to one item might determine the response to another item and therefore responses are not independent of each

For peer review only - http://bmjopen.bmj.com/site/about/guidelines.xhtml

other.[40] The residual correlation metric will identify if response dependency is an issue, with correlations <0.3.

Once the ‘Rasch factor’ is extracted, leftover residuals should not contain any patterns in the data.[40] A principal components analysis (PCA) of the residuals will detect if multi-dimensionality is an issue.[42] Subsets of items identified by the negative and positive correlations from the PCA will be used to compare estimates of responders’ health states on the two subsets. If no significant difference in the estimates is identified using independent t-test, then unidimensionality is assured. Tennant and Conaghan[40] also stated that the percentage of tests outside the range of -1.96 to 1.96 should not exceed 5%. A confidence level for the binomial test of proportions will be utilised for the number of significant tests, the lower bound should overlap the 5% expected value for a non-significant test.[40] If responders’ health state estimates are found to be significantly different in more than 5% of cases this will indicate that the subtests are measuring different but related aspects of health states.[40] Where the scale is being used to measure changes over time then using different but related subscales might be more appropriate.[80]

Finally, if the data fits the model, patient and item parameter estimates will be positioned on the same log-odds units (logits) scale, although as independent parameters allowing a linear transformation of the raw scores to be utilised.[80] Therefore, an estimate of a patients level of health can then be derived from the KDQOL-36 and IPOS-Renal data with confidence.

For peer review only - http://bmjopen.bmj.com/site/about/guidelines.xhtml

Sample size

There are various schools of thought regarding sample size requirements for validation studies.[82-85] We aim to recruit at least 180 patients based on the recommendations found in contemporary literature outlining psychometric best-practice (5 to 10 times the number of variables in any given multivariate statistical model).[82 83]

Reimbursement and Withdrawal

Reimbursement: All patients will be reimbursed for their time with a £20 gift card.[86 87] Light refreshments will be provided for focus group participants.

Withdrawal: Patient participants will be informed they have the right to freely withdraw from the study, for any reason, at any time prior to their data being integrated into the data set. They will not be required to supply a reason for their withdrawal and the decision will have no effect on their future medical care.

As focus group participants (clinicians and other professional staff) will be audio recorded as a group, they will be informed it will be impossible to withdraw their data during or after a focus group discussion. They will not be required to supply a reason for their withdrawal before a focus group discussion and the decision will have no effect on their employment.

For peer review only - http://bmjopen.bmj.com/site/about/guidelines.xhtml

DISCUSSION

PROMs can be completed electronically making it possible to remotely monitor symptoms in patients with CKD and generate ‘real time’ data which may assist clinicians with the tailoring of treatment to the needs of each patient.[12] The use of ePROMs could potentially foster patient-clinician communication and further support shared-decision between patients with CKD and their clinicians.[13 14] The regular completion of ePROMs may decrease the need for stable patients with acceptable ePROM data to attend clinical appointments thus sparing them the financial burden and physical stress of travelling. This might free up appointment times for patients who actually need to be seen in clinic.[12] In this manner, the use of PROMs may significantly improve the quality of life of patients with CKD.

At each stage of the project, when necessary, PAG meetings will be convened for their input and drafts of publication manuscripts reviewed by members of the group before submission to journals.

By employing a mixed methods approach, the PRO-trACK project will provide evidence of the feasibility and validity of the ePROM system in patients with advanced CKD.

For peer review only

For peer review only - http://bmjopen.bmj.com/site/about/guidelines.xhtml

ETHICS AND DISSEMINATION

Ethics and data management

This project was approved by the West Midlands Edgbaston Research Ethics Committee (Reference 17/WM/0010) and received HRA Approval on 24/02/2017. It has also been included in the National Institute for Health Research (NIHR) Clinical Research Network (CRN) Portfolio (ID 33117).

Participant data (whether in electronic or paper format) will be acquired, anonymised, transferred and stored according to the Data Protection Act 1998[88]; the Confidentiality - NHS Code of Practice[89]; the Caldicott principles[90]; the University of Birmingham Code of practice for research[91] and the University of Birmingham Guidance on Out of Hours Activities and Lone Working.[92]

Only members of the research team will have access to the project data. The exception will be permissions given to authorised regulatory personnel in order to conduct audits and inspections on behalf of the ethics committee.

Dissemination

The findings of the project will be provided to the Informatics Team and the Nephrology Unit at the QEHB as required. Participants will be given a summary of the findings, with reference to the full reports if desired.

Research article(s) based on the findings of the studies will be written and submitted for publication to peer-reviewed journals and all contributors and their contributions to the study will be acknowledged. We will also disseminate our findings at seminars and conferences both nationally and internationally.

For peer review only - http://bmjopen.bmj.com/site/about/guidelines.xhtml

Acknowledgement

The authors thank all the members of the Patient Advisory Group (PAG), Centre for Patient-Reported Outcomes Research (CPROR), University of Birmingham, for their comments and suggestions during the development of this project.

Author Contributions

MC is the guarantor for this project. The project was conceived by MC, DK, PC and TM and designed by MC, DK, PC, TM, OLA, MD and AS. OLA drafted the protocol manuscript. The manuscript was reviewed by MC, DK, PC, TM, OLA, MD, AS, NM, GP, RV, JW and KS. The final draft approved by all authors.

Funding

This project is funded as part of the Health Foundation's PhD Awards for Improvement Science. The Health Foundation is an independent charity working to improve the quality of healthcare in the UK. The Health Foundation was not involved in any other aspect of the project.

Tom Marshall is partly funded by the National Institute for Health Research (NIHR) through the Collaborations for Leadership in Applied Health Research and Care for West Midlands (CLAHRC-WM). This paper presents independent research and the views expressed in this publication are not necessarily those of the NIHR, the Department of Health, NHS Partner Trusts, University of Birmingham or the CLAHRC WM Management Group.

Competing interests: None declared.

For peer review only - http://bmjopen.bmj.com/site/about/guidelines.xhtml

Table 2. Definitions of domains, measurement properties, and aspects of measurement properties			
**Domain**	**Measurement property**	**Aspect of measurement property**	**Definition**
**Reliability**			The degree to which the measurement is free from measurement error
**Reliability (extended definition)**			The extent to which scores for patients who have not changed are the same for repeated measurement under several conditions: for example, using different sets of items from the same HR-PROs (internal consistency), over time (test-retest) by different persons on the same occasion (inter-rater) or by the same persons (i.e., raters or responders) on different occasions (intra-rater)
	Internal consistency		The degree of the interrelatedness among the items
	Reliability		The proportion of the total variance in the measurements which is because of "true"[a] differences among patients
	Measurement error		The systematic and random error of a patient's score that is not attributed to true changes in the construct to be measured
**Validity**			The degree to which an HR-PRO instrument measures the construct(s) it purports to measure

For peer review only - http://bmjopen.bmj.com/site/about/guidelines.xhtml

Table 2. Definitions of domains, measurement properties, and aspects of measurement properties			
**Domain**	**Measurement property**	**Aspect of measurement property**	**Definition**
	Content validity		The degree to which the content of an HR-PRO instrument is an adequate reflection of the construct to be measured
		Face validity	The degree to which (the items of) an HR-PRO instrument indeed looks as though they are an adequate reflection of the construct to be measured
	Construct validity		The degree to which the scores of an HR-PRO instrument are consistent with hypotheses (for instance with regard to internal relationships, relationships to scores of other instruments, or differences between relevant groups) based on the assumption that the HR-PRO instrument validly measures the construct to be measured
		Structural validity	The degree to which the scores of an HR-PRO instrument are an adequate reflection of the dimensionality of the construct to be measured
		Hypotheses testing	Idem construct validity
		Cross-cultural validity	The degree to which the performance of the items on a translated or culturally adapted HR-PRO instrument are an adequate reflection of the performance of the items of the original version of

For peer review only - http://bmjopen.bmj.com/site/about/guidelines.xhtml

Table 2. Definitions of domains, measurement properties, and aspects of measurement properties			
**Domain**	**Measurement property**	**Aspect of measurement property**	**Definition**
			the HR-PRO instrument
	Criterion validity		The degree to which the scores of an HR-PRO instrument are an adequate reflection of a ''gold standard''
**Responsiveness**			The ability of an HR-PRO instrument to detect change over time in the construct to be measured
	Responsiveness		Idem responsiveness
**Interpretability**[b]			The degree to which one can assign qualitative meaning - that is, clinical or commonly understood connotations - to an instrument's quantitative scores or change in scores.

Abbreviations: HR-PROs, health-related patient-reported outcomes; CTT, classical test theory.

a The word ''true'' must be seen in the context of the CTT, which states that any observation is composed of two components - a true score and error associated with the observation. ''True'' is the average score that would be obtained if the scale were given an infinite number of times. It refers only to the consistency of the score and not to its accuracy (ref Streiner & Norman [12]).

b Interpretability is not considered a measurement property but an important characteristic of a measurement instrument.

(Reproduced with permission from Caroline Terwee, COSMIN)

For peer review only

Bibliography

1. Levey AS, Coresh J. Chronic kidney disease. Lancet (London, England) 2012;**379**(9811):165-80.
2. K/DOQI clinical practice guidelines for chronic kidney disease: evaluation, classification, and stratification. American journal of kidney diseases : the official journal of the National Kidney Foundation 2002;**39**(2 Suppl 1):S1-266.
3. Meyer T, Hostetter, T. Medical Progress: Uremia. The New England journal of medicine 2007;**13**(357):1316-25.
4. Almutary H, Bonner A, Douglas C. Symptom burden in chronic kidney disease: a review of recent literature. Journal of Renal Care 2013;**39**(3):140-50.
5. Gapstur RL. Symptom burden: a concept analysis and implications for oncology nurses. Oncology nursing forum 2007;**34**(3):673-80.
6. Gansevoort RT, Matsushita K, van der Velde M, et al. Lower estimated GFR and higher albuminuria are associated with adverse kidney outcomes. A collaborative meta-analysis of general and high-risk population cohorts. Kidney international 2011;**80**(1):93-104.
7. Stringer S, Sharma P, Dutton M, et al. The natural history of, and risk factors for, progressive chronic kidney disease (CKD): the Renal Impairment in Secondary care (RIISC) study; rationale and protocol. BMC nephrology 2013;**14**:95.
8. FDA. Patient-reported outcome measures: use in medicinal product development to support labeling claims. Guidance for industry 2009. https://www.fda.gov/downloads/drugs/guidances/ucm193282.pdf (accessed Feb 2017).
9. Go AS, Chertow GM, Fan D, et al. Chronic Kidney Disease and the Risks of Death, Cardiovascular Events, and Hospitalization. New England Journal of Medicine 2004;**351**(13):1296-305.
10. Basch E, Bennett A, Pietanza MC. Use of patient-reported outcomes to improve the predictive accuracy of clinician-reported adverse events. Journal of the National Cancer Institute 2011;**103**(24):1808-10.
11. Bren L. 'The importance of patient-reported outcomes…It's all about the patients'. FDA Consumer 2006. https://permanent.access.gpo.gov/lps1609/www.fda.gov/fdac/features/2006/606_patients.html (accessed Feb 2017).
12. Hjollund NH, Larsen LP, Biering K, et al. Use of Patient-Reported Outcome (PRO) Measures at Group and Patient Levels: Experiences From the Generic Integrated PRO System, WestChronic. Interactive journal of medical research 2014;**3**(1):e5.
13. Velikova G, Booth L, Smith AB, et al. Measuring quality of life in routine oncology practice improves communication and patient well-being: a randomized controlled trial. Journal of clinical oncology : official journal of the American Society of Clinical Oncology 2004;**22**(4):714-24.
14. Gilbody SM, Whitty PM, Grimshaw JM, et al. Improving the detection and management of depression in primary care. Quality & safety in health care 2003;**12**(2):149-55.
15. Bennett AV, Jensen RE, Basch E. Electronic patient-reported outcome systems in oncology clinical practice. CA: a cancer journal for clinicians 2012;**62**(5):337-47.
16. McCann L, Maguire R, Miller M, et al. Patients' perceptions and experiences of using a mobile phone-based advanced symptom management system (ASyMS) to monitor and manage chemotherapy related toxicity. Eur J Cancer Care (Engl) 2009;**18**(2):156-64.
17. Aiyegbusi OL, Kyte D, Cockwell P, et al. Measurement properties of patient-reported outcome measures (PROMs) used in adult patients with chronic kidney disease: a systematic review protocol. BMJ Open 2016;**6**(10):e012014.
18. myhealth@QEHB University Hospitals Birmingham NHS Foundation Trust 2016. http://www.uhb.nhs.uk/myhealth-at-QEHB.htm (accessed Feb 2017).

For peer review only - http://bmjopen.bmj.com/site/about/guidelines.xhtml

19. EHI 2014 Awards. Digital Health 2014. http://www.ehilive.co.uk/ehi-awards/past-winners/2014-awards/best-use-of-technology-to-share-information-with-patients-and-carers/ (accessed Feb 2017).
20. Chao S, Yen M, Lin TC, et al. Psychometric Properties of the Kidney Disease Quality of Life-36 Questionnaire (KDQOL-36). West J Nurs Res 2016;**38**(8):1067-82.
21. Ricardo AC, Hacker E, Lora CM, et al. Validation of the Kidney Disease Quality of Life Short Form 36 (KDQOL-36TM) US Spanish and English versions in a cohort of Hispanics with chronic kidney disease. Ethnicity and Disease 2013;**23**(2):202-09.
22. POS. Palliative care Outcome Scale Website. https://pos-pal.org/ (accessed Feb 2017)
23. Johnson JA, Coons SJ, Ergo A, et al. Valuation of EuroQOL (EQ-5D) health states in an adult US sample. Pharmacoeconomics 1998;**13**(4):421-33.
24. Herdman M, Gudex C, Lloyd A, et al. Development and preliminary testing of the new five-level version of EQ-5D (EQ-5D-5L). Quality of Life Research 2011;**20**(10):1727-36.
25. Tangri N, Stevens LA, Griffith J, et al. A predictive model for progression of chronic kidney disease to kidney failure. Jama 2011;**305**(15):1553-9.
26. Jesky MD, Dutton M, Dasgupta I, et al. Health-Related Quality of Life Impacts Mortality but Not Progression to End-Stage Renal Disease in Pre-Dialysis Chronic Kidney Disease: A Prospective Observational Study. PloS one 2016;**11**(11):e0165675.
27. Usability.Gov. https://www.usability.gov/ (accessed Feb 2017).
28. Nielsen J. Usability Engineering. Academic Press Inc 1994:165.
29. Van Den Haak M, De Jong M, Jan Schellens P. Retrospective vs. concurrent think-aloud protocols: testing the usability of an online library catalogue. Behaviour & information technology 2003;**22**(5):339-51.
30. Terwee CB, Mokkink LB, Knol DL, et al. Rating the methodological quality in systematic reviews of studies on measurement properties: a scoring system for the COSMIN checklist. Quality of life research : an international journal of quality of life aspects of treatment, care and rehabilitation 2012;**21**(4):651-7.
31. Braun V, Clarke V. Using thematic analysis in psychology. Qualitative research in psychology 2006;**3**(2):77-101.
32. Bryman A. Social Research Methods. Oxford: Oxford University Press 2001.
33. Fusch Patricia NL. Are We There Yet? Data Saturation in Qualitative Research. The Qualitative Report 2015;**20**(9).
34. Rasch G. Probabilistic models for some intelligence and attainment tests. . Copenhagen: Danish Institute for Educational Research 1960.
35. Nunnally J. Psychometric Theory. McGraw-Hill, New York 1978.
36. Prieto L, Alonso J, Lamarca R. Classical Test Theory versus Rasch analysis for quality of life questionnaire reduction. Health and quality of life outcomes 2003;**1**:27.
37. Tennant A, Penta M, Tesio L, et al. Assessing and adjusting for cross-cultural validity of impairment and activity limitation scales through differential item functioning within the framework of the Rasch model: the PRO-ESOR project. Medical care 2004;**42**(1 Suppl):I37-48.
38. Yorke J, Horton M, Jones PW. A critique of Rasch analysis using the Dyspnoea-12 as an illustrative example. Journal of Advanced Nursing 2012;**68**(1):191-98.
39. Pallant JF, Tennant A. An introduction to the Rasch measurement model: an example using the Hospital Anxiety and Depression Scale (HADS). The British journal of clinical psychology 2007;**46**(Pt 1):1-18.
40. Tennant A, Conaghan PG. The Rasch measurement model in rheumatology: What is it and why use it? When should it be applied, and what should one look for in a Rasch paper? Arthritis Care & Research 2007;**57**(8):1358-62.
41. Wright BD. Solving Measurement Problems with the Rasch Model. Journal of Educational Measurement 1977;**14**(2):97-116.

For peer review only - http://bmjopen.bmj.com/site/about/guidelines.xhtml

42. Smith EV. Detecting and evaluation the impact of multidimensionality using item fit statistics and principal component analysis of residuals. Journal Applied Measurement 2002;**3**:205-31.
43. de Vet HC, Ader HJ, Terwee CB, et al. Are factor analytical techniques used appropriately in the validation of health status questionnaires? A systematic review on the quality of factor analysis of the SF-36. Quality of life research : an international journal of quality of life aspects of treatment, care and rehabilitation 2005;**14**(5):1203-18; dicussion 19-21, 23-4.
44. Dexter PR, Stump TE, Tierney WM, et al. The psychometric properties of the SF-36 health survey among older adults in a clinical setting. Journal of Clinical Geropsychology 1996;**2**(3):225-37.
45. Kaiser HF. The Application of Electronic Computers to Factor Analysis. Educational and Psychological Measurement 1960;**20**(1):141-51.
46. Cattell RB. The Scree Test For The Number Of Factors. Multivariate Behavioral Research 1966;**1**(2):245-76.
47. Wolinsky FD, Stump TE. A measurement model of the Medical Outcomes Study 36-Item Short-Form Health Survey in a clinical sample of disadvantaged, older, black, and white men and women. Medical care 1996;**34**(6):537-48.
48. Cronbach LJ. Coefficient alpha and the internal structure of tests. Psychometrika 1951;**16**(3):297-334.
49. Streiner DL. Starting at the beginning: an introduction to coefficient alpha and internal consistency. Journal of personality assessment 2003;**80**(1):99-103.
50. Tavakol M, Dennick R. Making sense of Cronbach's alpha. International Journal of Medical Education 2011;**2**:53-55.
51. Nunnally JC, Bernstein IH. Psychometric theory. New York: McGraw-Hill 1994;**3rd edition**.
52. Gliem JA, Gliem RR. Calculating, Interpreting, and Reporting Cronbach's Alpha Reliability Coefficient for Likert-Type Scales. 2003.
53. Shrout PE, Fleiss JL. Intraclass correlations: uses in assessing rater reliability. Psychological bulletin 1979;**86**(2):420-8.
54. Terwee CB, Bot SD, de Boer MR, et al. Quality criteria were proposed for measurement properties of health status questionnaires. Journal of clinical epidemiology 2007;**60**(1):34-42.
55. McGraw KO, Wong SP. Forming inferences about some intraclass correlation coefficients. Psychological Methods 1996;**1**(1):30-46.
56. Bataclan RP, Dial MAD. Cultural adaptation and validation of the Filipino version of Kidney Disease Quality of Life - Short Form (KDQOL-SF version 1.3). Nephrology 2009;**14**(7):663-68.
57. Stratford P. Reliability: consistency or differentiating among subjects? Phys Ther 1989;**69**(4):299-300.
58. Guyatt GH, Osoba D, Wu AW, et al. Methods to Explain the Clinical Significance of Health Status Measures. Mayo Clin Proc;**77**(4):371-83.
59. Jaeschke R, Singer J, Guyatt GH. Measurement of health status. Ascertaining the minimal clinically important difference. Control Clin Trials 1989;**10**(4):407-15.
60. Kamper SJ, Maher CG, Mackay G. Global Rating of Change Scales: A Review of Strengths and Weaknesses and Considerations for Design. The Journal of Manual & Manipulative Therapy 2009;**17**(3):163-70.
61. Johnston BC, Ebrahim S, Carrasco-Labra A, et al. Minimally important difference estimates and methods: a protocol. BMJ Open 2015;**5**(10).
62. Norman GR, L. SD. Biostatistics: the bare essentials. . St Louis, MO: Mosby 1994.
63. Pakpour AH, Nourozi S, Molsted S, et al. Validity and reliability of short form-12 questionnaire in Iranian hemodialysis patients. Iran J Kidney Dis 2011;**5**(3):175-81.
64. Ware JE, Jr., Gandek B. Methods for testing data quality, scaling assumptions, and reliability: the IQOLA Project approach. International Quality of Life Assessment. Journal of clinical epidemiology 1998;**51**(11):945-52.

For peer review only

For peer review only - http://bmjopen.bmj.com/site/about/guidelines.xhtml

65. Yang F, Wang VW, Joshi VD, et al. Validation of the English version of the Kidney Disease Quality of Life questionnaire (KDQOL-36) in haemodialysis patients in Singapore. Patient 2013;**6**(2):135-41.
66. Park HJ, Kim S, Yong JS, et al. Reliability and validity of the Korean version of kidney disease quality of life instrument (KDQOL-SFTM). Tohoku Journal of Experimental Medicine 2007;**211**(4):321-29.
67. Tao X, Chow SK, Wong FK. Determining the validity and reliability of the Chinese version of the Kidney Disease Quality of Life Questionnaire (KDQOL-36). BMC nephrology 2014;**15**:115.
68. Korevaar JC, Merkus MP, Jansen MAM, et al. Validation of the KDQOL-SFTM: A dialysis-targeted health measure. Quality of Life Research 2002;**11**(5):437-47.
69. Thaweethamcharoen T, Srimongkol W, Noparatayaporn P, et al. Validity and reliability of KDQOL-36 in thai kidney disease patient. Value in Health Regional Issues 2013;**2**(1):98-102.
70. Mokkink LB, Terwee CB, Knol DL, et al. The COSMIN checklist for evaluating the methodological quality of studies on measurement properties: a clarification of its content. BMC Med Res Methodol 2010;**10**:22.
71. Cheung YB, Seow YY, Qu LM, et al. Measurement properties of the Chinese version of the Kidney Disease Quality of Life-Short Form (KDQOL-SFTM) in end-stage renal disease patients with poor prognosis in singapore. Journal of Pain and Symptom Management 2012;**44**(6):923-32.
72. Kazis LE, Anderson JJ, Meenan RF. Effect sizes for interpreting changes in health status. Medical care 1989;**27**(3 Suppl):S178-89.
73. Liang MH, Fossel AH, Larson MG. Comparisons of five health status instruments for orthopedic evaluation. Medical care 1990;**28**(7):632-42.
74. Cohen J. Statistical power analysis for behavioral sciences. New York: Academic Press 1977.
75. Miller KJ, Slade AL, Pallant JF, et al. Evaluation of the psychometric properties of the upper limb subscales of the Motor Assessment Scale using a Rasch analysis model. Journal of Rehabilitation Medicine 2010;**42**(4):315-22.
76. RUMM2030 [program]. Perth, Australia: RUMM Laboratory Pty Ltd 2010.
77. Linacre JM. Optimizing rating scale category effectiveness. Journal of applied measurement 2002;**3**(1):85-106.
78. Andrich D. An Expanded Derivation of the Threshold Structure of the Polytomous Rasch Model That Dispels Any “Threshold Disorder Controversy”. Educational and Psychological Measurement 2013;**73**(1):78-124.
79. Hagquist C, Bruce M, Gustavsson JP. Using the Rasch model in nursing research: An introduction and illustrative example. International Journal of Nursing Studies 2009;**46**(3):380-93.
80. Pallant JF, Tennant A. An introduction to the Rasch measurement model: An example using the Hospital Anxiety and Depression Scale (HADS). British Journal of Clinical Psychology 2007;**46**:1-18.
81. Tennant A, Penta M, Tesio L, et al. Assessing and adjusting for cross cultural validity of impairment and activity limitation scales through Differential Item Functioning within the framework of the Rasch model : The Pro-ESOR project Medical Care 2004;**42**:37-48.
82. Tabachnik BJ, Fidel LS. Using multivariate statistics. London: Harper & Row 1993.
83. Terwee CB. Protocol for systematic reviews of measurement properties. COSMIN 2011. http://www.cosmin.nl/images/upload/files/Protocol%20klinimetrische%20review%20versio n%20nov%202011.pdf (accessed Feb 2017).
84. Velicer WF, Fava JL. Affects of variable and subject sampling on factor pattern recovery. Psychological Methods 1998;**3**(2):231-51.
85. MacCallum RC, Widaman KF, Zhang S, et al. Sample size in factor analysis. Psychological Methods 1999;**4**(1):84-99.
86. INVOLVE. Payment for Involvement - A guide for making payments to members of the public actively involved in NHS, public health and social care research. NIHR 2010.

For peer review only - http://bmjopen.bmj.com/site/about/guidelines.xhtml

http://www.invo.org.uk/wp-content/uploads/2012/11/INVOLVEPayment-Guiderev2012.pdf (accessed Feb 2017).
87. Draper H, Wilson S, Flanagan S, et al. Offering payments, reimbursement and incentives to patients and family doctors to encourage participation in research. Family practice 2009;**26**(3):231-38.
88. Parliament of the United Kingdom of Great Britain and Northern Ireland. Data Protection Act 1998. 2005:92. http://www.legislation.gov.uk/ukpga/1998/29/pdfs/ukpga_19980029_en.pdf (accessed Feb 2017).
89. DOH. Confidentiality - NHS Code of Practice. Department of Health 2003. https://www.gov.uk/government/publications/confidentiality-nhs-code-of-practice (accessed Feb 2017).
90. DOH. The Caldicott Committee. Report on the Review of Patient-Identifiable Information Department of Health 1997. http://webarchive.nationalarchives.gov.uk/+/www.dh.gov.uk/en/Publicationsandstatistics/Publications/PublicationspolicyandGuidance/DH_4068403 (accessed Feb 2017).
91. UoB. Code of practice for research. University of Birmingham 2015 - 2016. http://www.birmingham.ac.uk/Documents/university/legal/research.pdf (accessed Feb 2017).
92. UoB. Guidance on out of hours activities and lone working. University of Birmingham 2012. https://intranet.birmingham.ac.uk/hr/documents/public/hsu/hsuguidance/31ohalw.pdf (accessed Feb 2017).

For peer review only

For peer review only - http://bmjopen.bmj.com/site/about/guidelines.xhtml

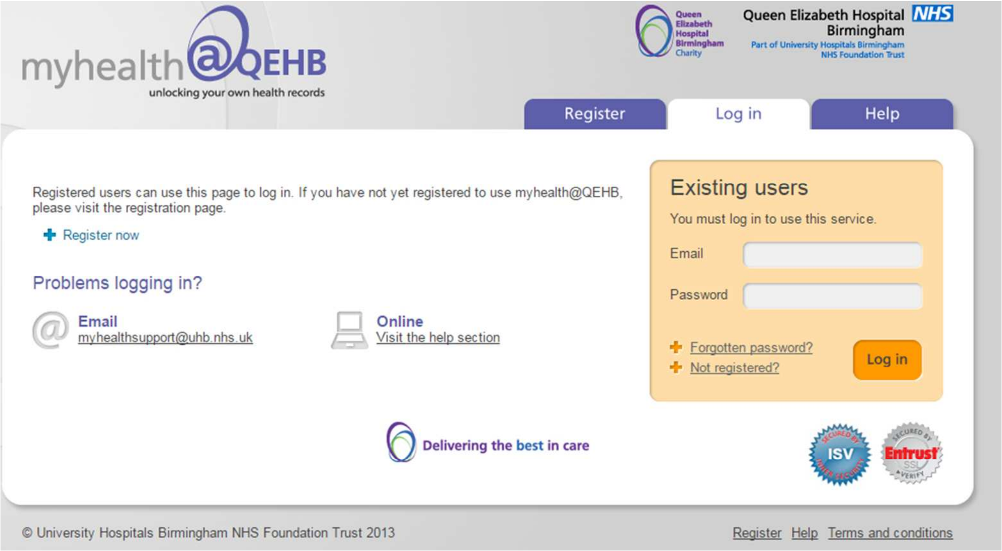

Figure 1. Screenshot of the myhealth@QEHB login page

83x45mm (300 x 300 DPI)

review only
